# Preclinical comparison of proteasome and ubiquitin E1 enzyme inhibitors in cutaneous squamous cell carcinoma: the identification of mechanisms of differential sensitivity

**DOI:** 10.18632/oncotarget.24750

**Published:** 2018-04-17

**Authors:** Angela McHugh, Kenneth Fernandes, Andrew P. South, Jemima E. Mellerio, Julio C. Salas-Alanís, Charlotte M. Proby, Irene M. Leigh, Mark K. Saville

**Affiliations:** ^1^ Division of Cancer Research, School of Medicine, University of Dundee, Dundee DD1 9SY, UK; ^2^ Department of Dermatology and Cutaneous Biology, Thomas Jefferson University, Philadelphia, PA 19107, USA; ^3^ St. John’s Institute of Dermatology, King’s College London, Guy’s Campus, London, SE1 7EH, UK; ^4^ DEBRA Mexico, Azteca Guadalupe, Nuevo Leon, 67150 Mexico; ^5^ Hospital Regional “Lic. Adolfo Lopez Mateos”, Colonia Florida, Del Alvaro Obregon, 01030 Ciudad de Mexico; ^6^ Centre for Cutaneous Research, Barts and the London School of Medicine and Dentistry, Queen Mary University of London, London E1 2AT, UK

**Keywords:** squamous cell carcinoma, proteasome, MLN7243/TAK-243, UBA1, UBA6

## Abstract

Proteasome inhibitors have distinct properties and the biochemical consequences of suppressing ubiquitin E1 enzymes and the proteasome differ. We compared the effects of the proteasome inhibitors bortezomib, ixazomib and carfilzomib and the ubiquitin E1 enzyme inhibitor MLN7243/TAK-243 on cell viability and cell death in normal keratinocytes and cutaneous squamous cell carcinoma (cSCC) cell lines. The effects of both a pulse of treatment and more extended incubation were investigated. This is relevant to directly-delivered therapy (topical treatment/intratumoral injection) where the time of exposure can be controlled and a short exposure may better reflect systemically-delivered inhibitor pharmacokinetics. These agents can selectively kill cSCC cells but there are variations in the pattern of cSCC cell line sensitivity/resistance. Variations in the responses to proteasome inhibitors are associated with differences in the specificity of the inhibitors for the three proteolytic activities of the proteasome. There is greater selectivity for killing cSCC cells compared to normal keratinocytes with a pulse of proteasome inhibitor treatment than with a more extended exposure. We provide evidence that c-MYC-dependent NOXA upregulation confers susceptibility to a short incubation with proteasome inhibitors by priming cSCC cells for rapid BAK-dependent death. We observed that bortezomib-resistant cSCC cells can be sensitive to MLN7243-induced death. Low expression of the ubiquitin E1 UBA1/UBE1 participates in conferring susceptibility to MLN7243 by increasing sensitivity to MLN7243-mediated attenuation of ubiquitination. This study supports further investigation of the potential of proteasome and ubiquitin E1 inhibition for cSCC therapy. Direct delivery of inhibitors could facilitate adequate exposure of skin cancers.

## INTRODUCTION

Cutaneous squamous cell carcinoma (cSCC) causes significant mortality and morbidity in the general population [1]. In addition, cSCC has a huge impact on patients with the chronic skin blistering disease recessive dystrophic epidermolysis bullosa (RDEB) [2]. The cumulative risk of death from cSCC in patients with severe generalized RDEB is 80% by the age of 55 [3]. Immunosuppressed patients are also at high risk of cSCC. For example, organ transplant recipients are up to 150-fold more likely to develop cSCC than the general population and these tumours tend to be more aggressive [4]. cSCC is responsible for 25% of deaths due to skin cancer in the United Kingdom and in some regions of the United States it is responsible for more deaths than melanoma [1]. Most cSCCs can be successfully treated by surgery. However, there is a need for improved therapy for the minority of cSCCs that are responsible for this substantial health burden [5].

Ubiquitination regulates protein stability, activity, association and localization [6, 7]. This is important in many fundamental processes and pathways of relevance to tumorigenesis and cancer therapy. Ubiquitination of target proteins involves three classes of enzymes. Ubiquitin is loaded onto ubiquitin conjugating enzymes (E2s) by ubiquitin activating enzymes (E1s). Ubiquitin ligases (E3s) facilitate transfer of ubiquitin from E2s to substrates [8, 9]. There are two ubiquitin E1s in the human genome: UBA1/UBE1 and UBA6 [10-12]. These E1s pass ubiquitin onto overlapping but distinct groups of E2s [11, 13]. UBA1 is thought to be involved in ubiquitin conjugation to the majority of cellular substrates [11]. However, UBA6 is required for the ubiquitination of a subset of proteins [13]. UBA6 is also the E1 responsible for activation of the ubiquitin-like (UBL) protein FAT10 [10, 14]. Ubiquitination can target proteins for proteasomal degradation. The proteasome is formed from two multi-subunit protein complexes: the 20S core and the 19S regulatory particle. It has three proteolytic activities which are conferred by different subunits in its 20S core: PSMB6/β1 (caspase-like), PSMB7/β2 (trypsin-like) and PSMB5/β5 (chymotrypsin-like). Protein degradation is differentially dependent on these proteolytic activities [15, 16]. The chymotrypsin-like activity plays a major role in protein degradation but the other activities of the proteasome make an additional contribution.

The proteasome inhibitor bortezomib has been widely used to treat patients with multiple myeloma. Two additional proteasome inhibitors: ixazomib and carfilzomib have recently been approved for multiple myeloma therapy. These agents differ in their affinity for inhibition of the individual proteolytic activities of the proteasome and/or in their reversibility. The importance of these differences in influencing the therapeutic efficacy of these inhibitors is not fully understood. At low concentrations all of these compounds inhibit the chymotrypsin-like activity of the proteasome. Bortezomib and ixazomib inhibit the caspase-like activity at moderately high concentrations and the trypsin-like activity at high concentrations [17]. Moderately high concentrations of carfilzomib inhibit both the caspase and trypsin-like activities [18, 19]. In cells the half-life for recovery of the chymotrypsin-like activity of the proteasome after inhibitor removal is less than 4 hours for ixazomib, 15 to 20 hours for bortezomib and can be less than 24 hours for carfilzomib [18-21]. Following intravenous injection proteasome inhibitors reach μM levels in plasma but the concentration declines within hours [22-24]. Binding to proteasomes in patient tissues could contribute to this reduction in levels [25]. There is a secondary slower phase of elimination and low plasma concentrations of proteasome inhibitors can be maintained for several days. Effective systemic treatment is required for distant cSCC metastasis [26]. There are ongoing clinical trials in other cancers, including trials involving the more recently developed inhibitors, but to date the response of solid tumours in general to systemic proteasome inhibitor therapy has been poor [23, 27]. This may be related to difficulties in achieving a sufficient extent or duration of proteasome inhibition in solid tumours by systemic delivery [16, 28]. However, there is great potential for treating skin cancer including cSCC by topical therapy or intratumoral injection. With directly-delivered therapy the inhibitor dose and the duration of inhibitor exposure could be controlled to achieve optimum tumour selectivity [28-31]. Patients with inoperable loco-regional disease would benefit from effective directly-delivered drug therapy [32-34]. It would also be useful in neoadjuvant and adjuvant settings to reduce the impact of surgery and to prevent local tumour recurrence [32, 35, 36].

An alternative way of targeting the core ubiquitin-proteasome system is to inhibit the ubiquitin E1 enzymes [37]. Blocking ubiquitin conjugation by suppressing ubiquitin E1s has the potential to attenuate all cellular events that are controlled by ubiquitination not simply proteasomal degradation. Substrate ubiquitination can however be differentially dependent on the level of E1 activity [13, 38]. The cellular pathways affected may consequently be determined by the extent of E1 inhibition. MLN7243/TAK-243 is a recently developed high-affinity inhibitor with selectivity for ubiquitin E1s over other UBL protein E1s [39-42]. We confirm in this report that it can suppress both UBA1 and UBA6. MLN7243 is the first ubiquitin E1 inhibitor to enter a clinical trial for cancer therapy (NCT02045095). It has been observed that in mice systemically treated with MLN7243 plasma levels decline rapidly but the inhibitor has a long half-life in tumours [39].

The therapeutic potential for cSCC of targeting the ubiquitin-proteasome system has not been adequately explored. Furthermore, the anti-tumour activity and the degree of tumour selectivity of proteasome and ubiquitin E1 inhibition have not been compared for any cancer type. It is common in pre-clinical investigations to determine the effect of extended exposure to proteasome inhibitors on the viability of cultured cells. It is relevant for directly-delivered therapy to assess the effects of different times of tumour treatment. It may also better model systemically-delivered inhibitor pharmacokinetics to investigate the consequences of a shorter pulse of inhibitor treatment [21-24, 39]. Multiple pathways have been implicated in killing cells in response to continuous proteasome inhibitor exposure [43]. Much remains to be learned about the dominant mechanisms underlying sensitivity to a pulse of proteasome inhibition. In addition, little is known regarding the determinants of sensitivity to ubiquitin E1 suppression. In this study we compared the effects on cell viability and cell death of long and short exposure to the three clinically approved proteasome inhibitors and the ubiquitin E1 inhibitor MLN7243. This was done in normal keratinocytes and in cSCC cell lines derived from RDEB, immunocompetent and immunosuppressed patients. These agents can selectively kill cSCC cells originating from primary and metastatic tumours. There is greater selectivity for cSCC compared to normal keratinocytes with a pulse of proteasome inhibitor exposure than with a more extended treatment. There are differences between inhibitors in the magnitude of the window of tumour selectivity and in the pattern of sensitivity of cSCC cell lines. Mechanisms contributing to these differences were identified.

## RESULTS

### Comparison of the anti-cSCC activity of proteasome and ubiquitin E1 inhibitors

**Figure 1:**
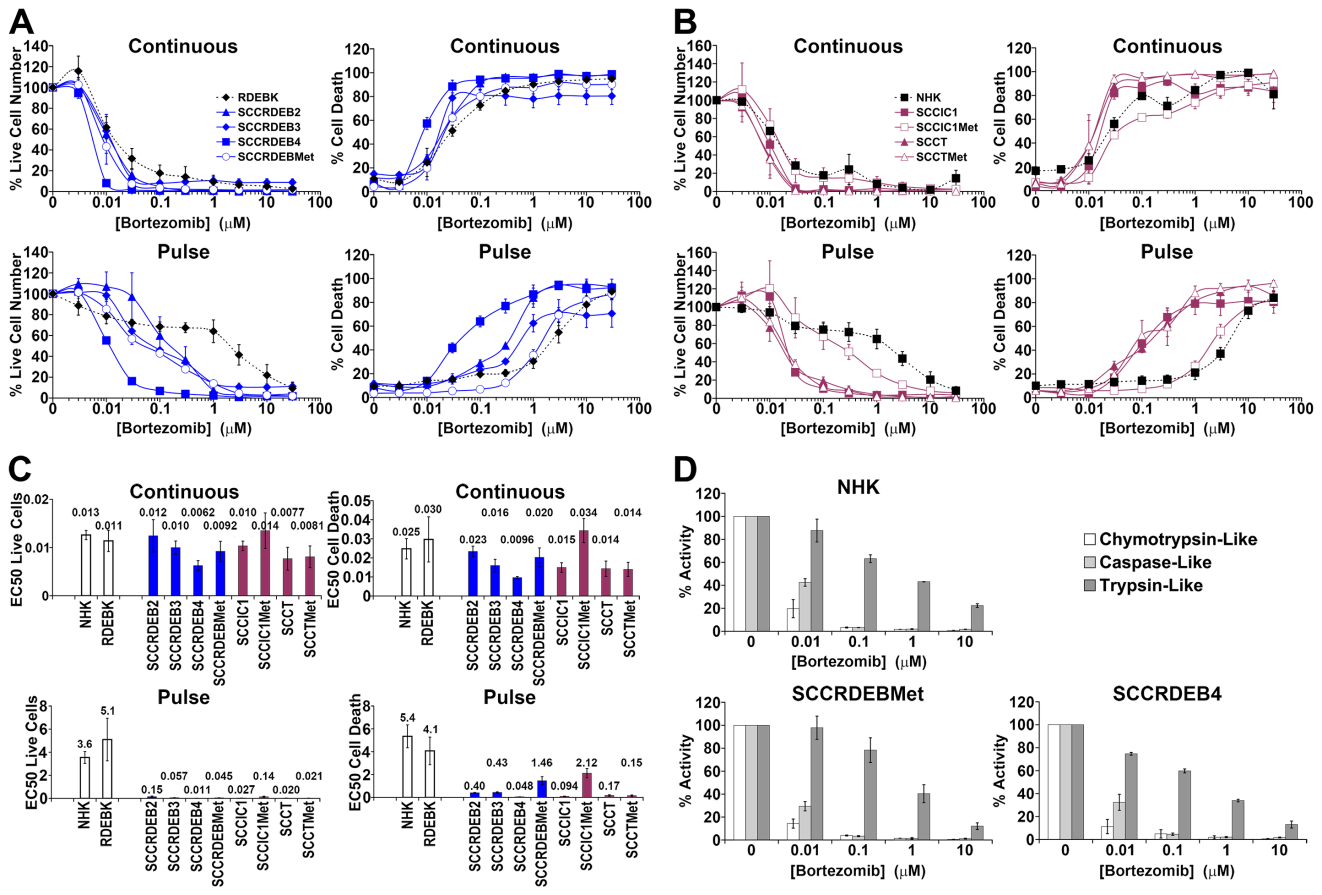
cSCC cell lines can be highly sensitive to a pulse of bortezomib **(A-C)** Cells were continuously incubated with the proteasome inhibitor bortezomib for 72 hours (Continuous) or treated with bortezomib for 8 hours and then maintained in drug-free medium for a further 64 hours (Pulse). Cell viability (live cell number) expressed as a percentage of carrier alone and the percentage of dead cells were assayed by real-time imaging. Values are the mean -/+ SEM of 3 independent experiments. (A) Normal keratinocytes from an RDEB patient (RDEBK) and cSCC cell lines derived from primary tumours from RDEB patients (SCCRDEB 2, 3 and 4) and from an RDEB cSCC metastasis (SCCRDEBMet). (B) Normal human keratinocytes (NHK) and cSCC lines derived from paired primary tumours and metastases from immunocompetent (SCCIC1 and SCCIC1Met) and transplant patients (SCCT and SCCTMet). (C) Relative EC50 values (μM) for reducing cell viability (live cell number) and for promoting cell death. There was little cSCC selectivity with continuous exposure to bortezomib but the majority of cSCC cell lines were more sensitive to a pulse of bortezomib than normal keratinocytes. **(D)** The three proteolytic activities of the proteasome were assayed 8 hours after the addition of bortezomib. The results were expressed as a percentage of the activity with carrier alone. Values are the mean -/+ SEM of 3 independent experiments. The cSCC cells lines most sensitive to pulse of bortezomib were killed at bortezomib concentrations that strongly inhibit both the chymotrypsin and caspase-like activities of the proteasome.

**Figure 2:**
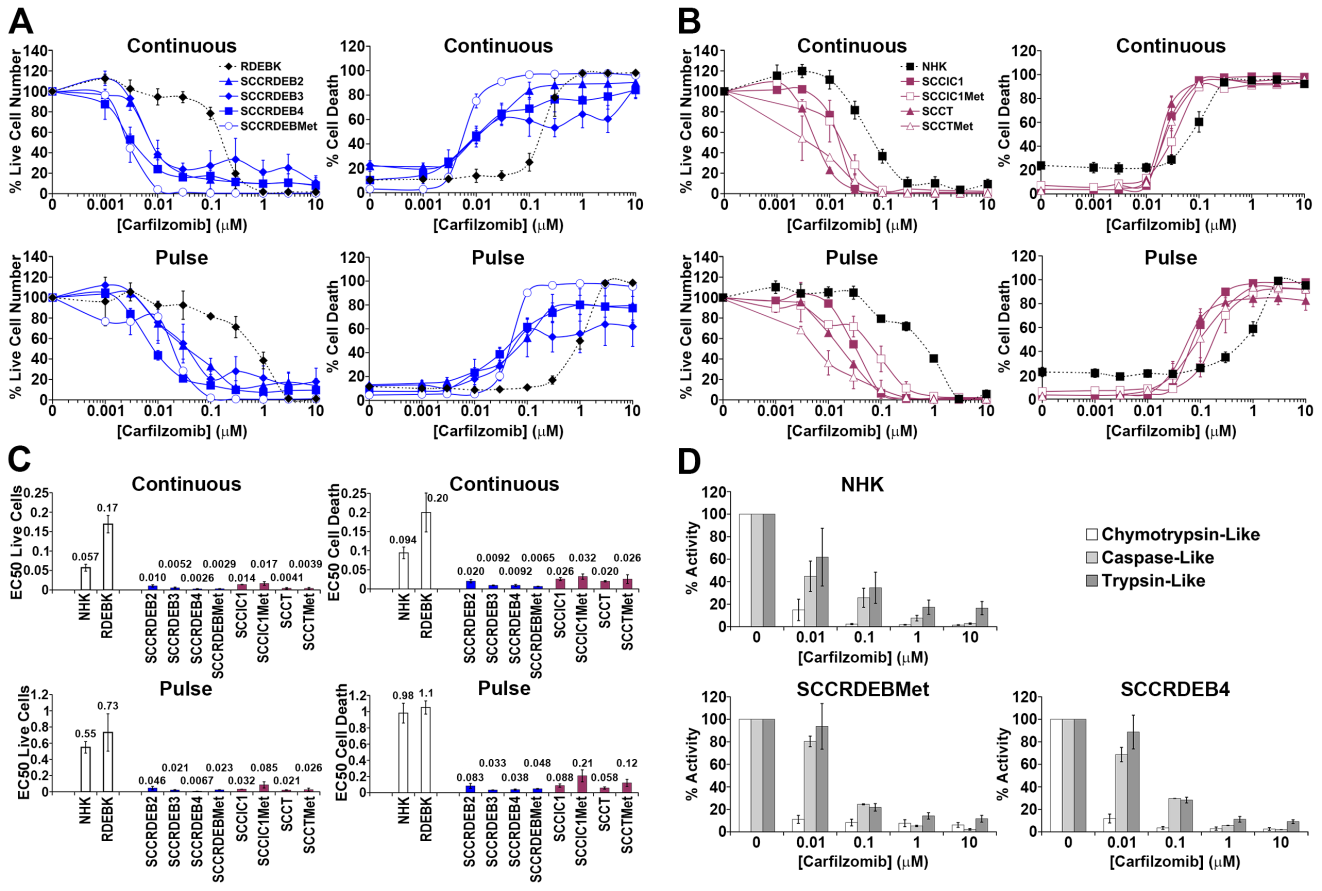
Carfilzomib exhibits more general anti-cSCC selectivity than bortezomib **(A-C)** Cells were continuously incubated with the proteasome inhibitor carfilzomib or treated with an 8-hour carfilzomib pulse. Cell viability (live cell number), expressed as a percentage of carrier alone and the percentage of dead cells were assayed by real-time imaging 72 hours after drug addition. Values are the mean -/+ SEM of 3 independent experiments. (A) Normal keratinocytes and cSCC cell lines from RDEB patients. (B) Normal keratinocytes and cSCC cell lines from: immunocompetent (SCCIC) and transplant (SCCT) patients. (C) Relative EC50 values (μM) for reducing cell viability (live cell number) and for promoting cell death. cSCC cell lines were more sensitive to continuous carfilzomib treatment and a pulse of carfilzomib exposure than normal keratinocytes. **(D)** The proteolytic activities of the proteasome were assayed 8 hours after the addition of carfilzomib. The results were expressed as a percentage of the activity with carrier alone. Values are the mean -/+ the range of 2 independent experiments. Cell death in RDEB cSCC cell lines in response to continuous exposure to carfilzomib occurred at carfilzomib concentrations where only the chymotrypsin-like activity was inhibited. Death of cSCC cells induced by a short pulse of carfilzomib was associated with robust inhibition of the chymotrypsin-like activity and moderate inhibition of both the caspase and trypsin-like activities.

**Figure 3:**
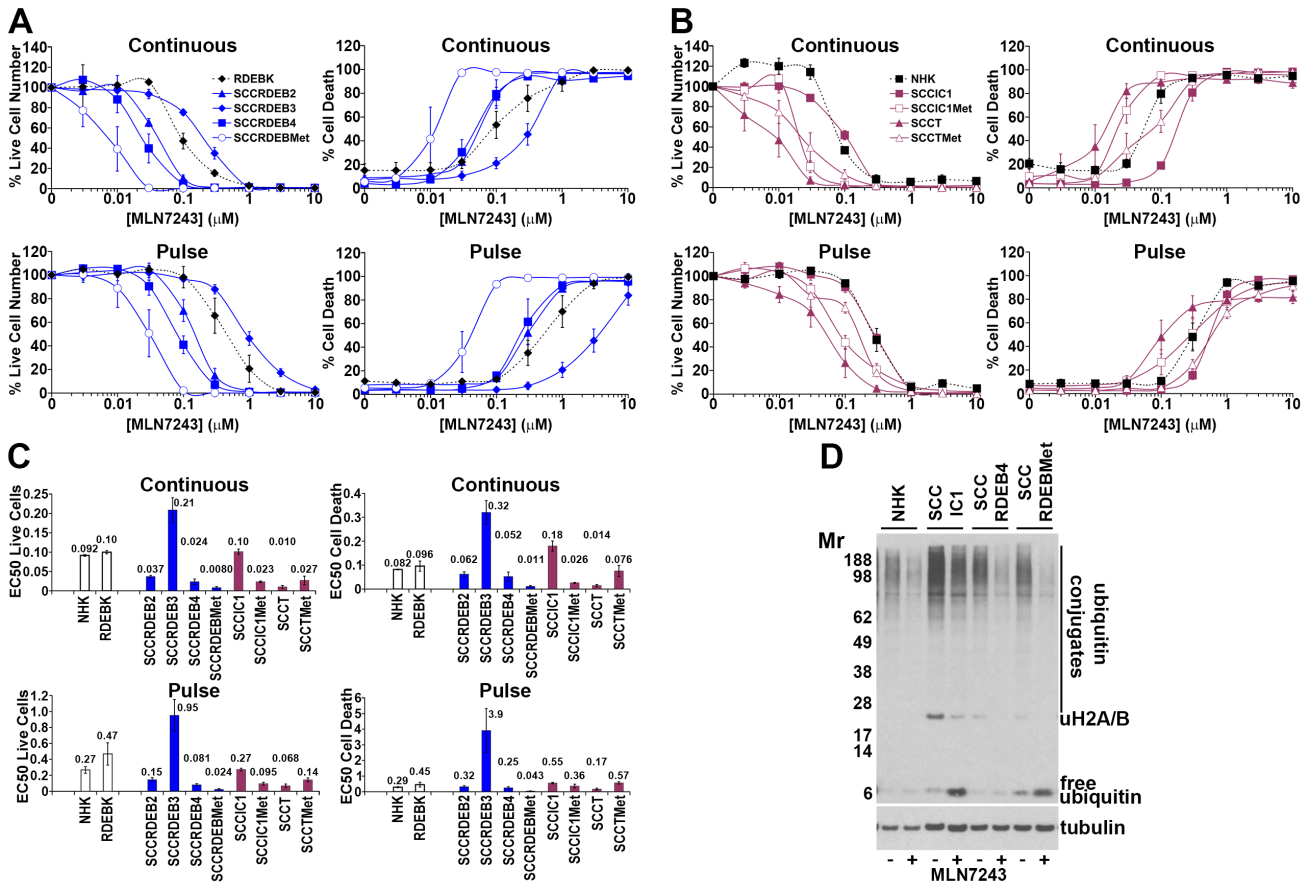
Bortezomib-resistant cSCC cells can be sensitive to MLN7243 **(A-C)** Cells were continuously incubated with the ubiquitin E1 inhibitor MLN7243 or treated with an 8-hour MLN7243 pulse. Cell viability (live cell number), expressed as a percentage of carrier alone and the percentage of dead cells were assayed by real-time imaging 72 hours after drug addition. Values are the mean -/+ SEM of 3 independent experiments. (A) Normal keratinocytes and cSCC cell lines from RDEB patients. (B) Normal keratinocytes and cSCC cell lines from: immunocompetent (SCCIC) and transplant (SCCT) patients. (C) Relative EC50 values (μM) for reducing cell viability (live cell number) and for promoting cell death. SCCT cells and bortezomib-resistant SCCRDEBMet and SCCIC1Met cells were sensitive to death induced by MLN7243. **(D)** Cells were treated with carrier (-) or 0.1μM MLN7243 (+) for 12 hours and analysed by western blotting. Consistent with ubiquitin E1 inhibition MLN7243 reduced high molecular weight ubiquitin conjugates and ubiquitinated histone H2A/H2B and increased free ubiquitin.

We investigated whether there were differences in responses to the proteasome inhibitors bortezomib, ixazomib and carfilzomib and the ubiquitin E1 inhibitor MLN7243. The effects of these agents were assessed in untransformed normal keratinocytes from an RDEB patient (RDEBK), normal human keratinocytes (NHK) and eight cSCC-derived cell lines. cSCCs arise from the malignant transformation of keratinocytes in the epidermis. Keratinocytes constitute over 90% of cells within this layer of the skin. It is consequently appropriate to include keratinocytes in the study as the cell of cSCC origin and a major normal cell type that would be exposed to locally-delivered therapy. Measurement of the proteolytic activities of the proteasome verified target suppression by proteasome inhibitors (Figure 1D and 2D and Supplementary Figure 3D). In addition, we confirmed that MLN7243 can reduce the cellular level of ubiquitin conjugates (Figure 3D). As part of the catalytic mechanism E1 and E2 enzymes form reducing agent-sensitive thioesters with ubiquitin and UBL proteins [41, 44]. MLN7243 decreased UBA1 and UBA6 thioesters and thioesters of the UBA6 specific E2 UBE2Z/USE1 (Supplementary Figure 1 and Figure 7). This confirms that both UBA1 and UBA6 are inhibited by MLN7243.

**Figure 4:**
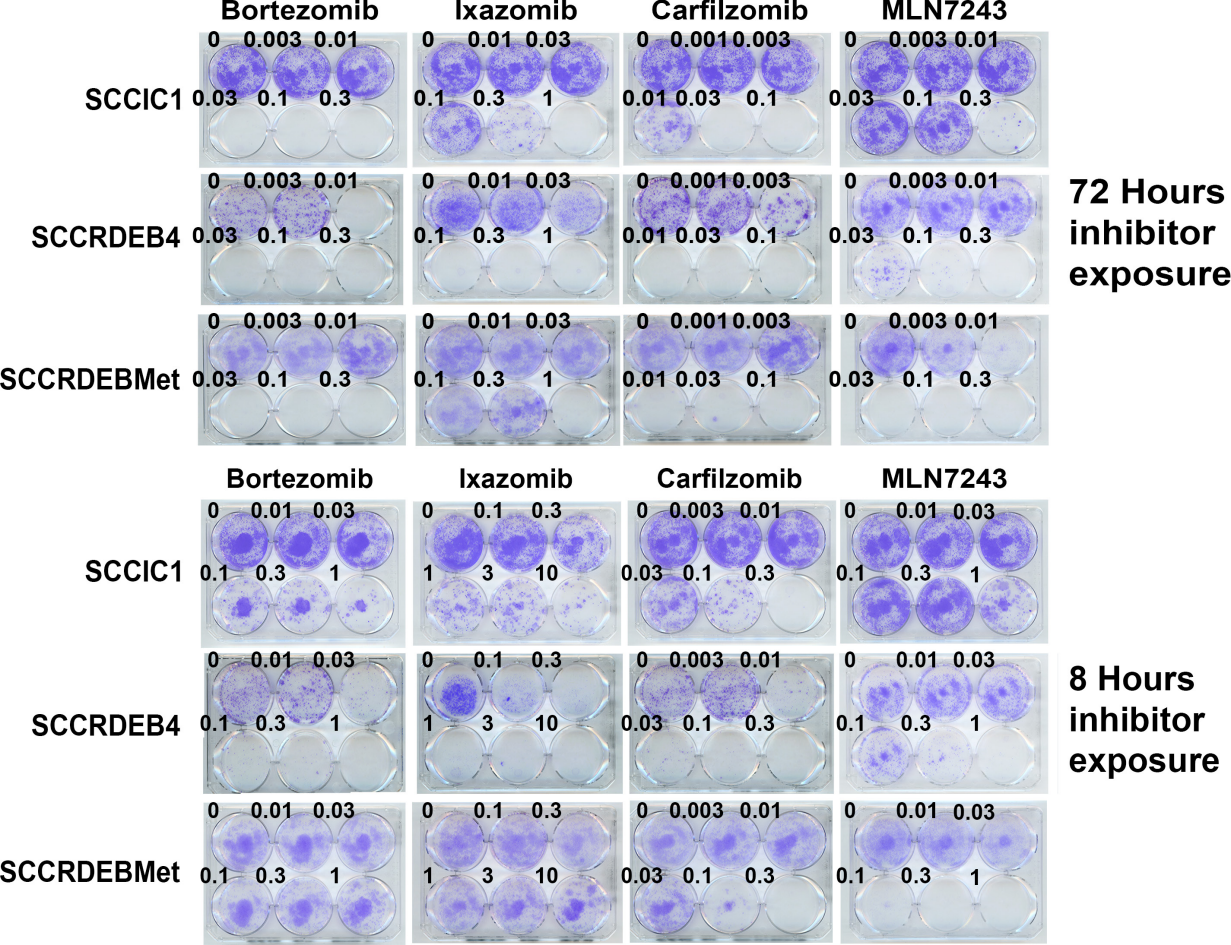
Differences in cSCC cell line sensitivity to proteasome and ubiquitin E1 inhibitors are recapitulated in clonogenic assays Cells were treated with inhibitors for 72 or 8 hours, comparable to the previously used extended exposure and pulse respectively. Cells were then maintained in drug-free medium to allow colony formation. The pattern of inhibitor sensitivity was similar to that observed using shorter-term real-time imaging assays. SCCIC1 cells were relatively sensitive to proteasome inhibitors but resistant to the ubiquitin E1 inhibitor MLN7243. SCCRDEB4 cells are highly sensitive to proteasome inhibitors and intermediately sensitive to MLN7243. SCCRDEBMet cells were insensitive to an 8-hour treatment with bortezomib or ixazomib but sensitive to an 8-hour exposure to carfilzomib. SCCRDEBMet cells were sensitive to 8 and 72 hours treatment with MLN7243.

**Figure 5:**
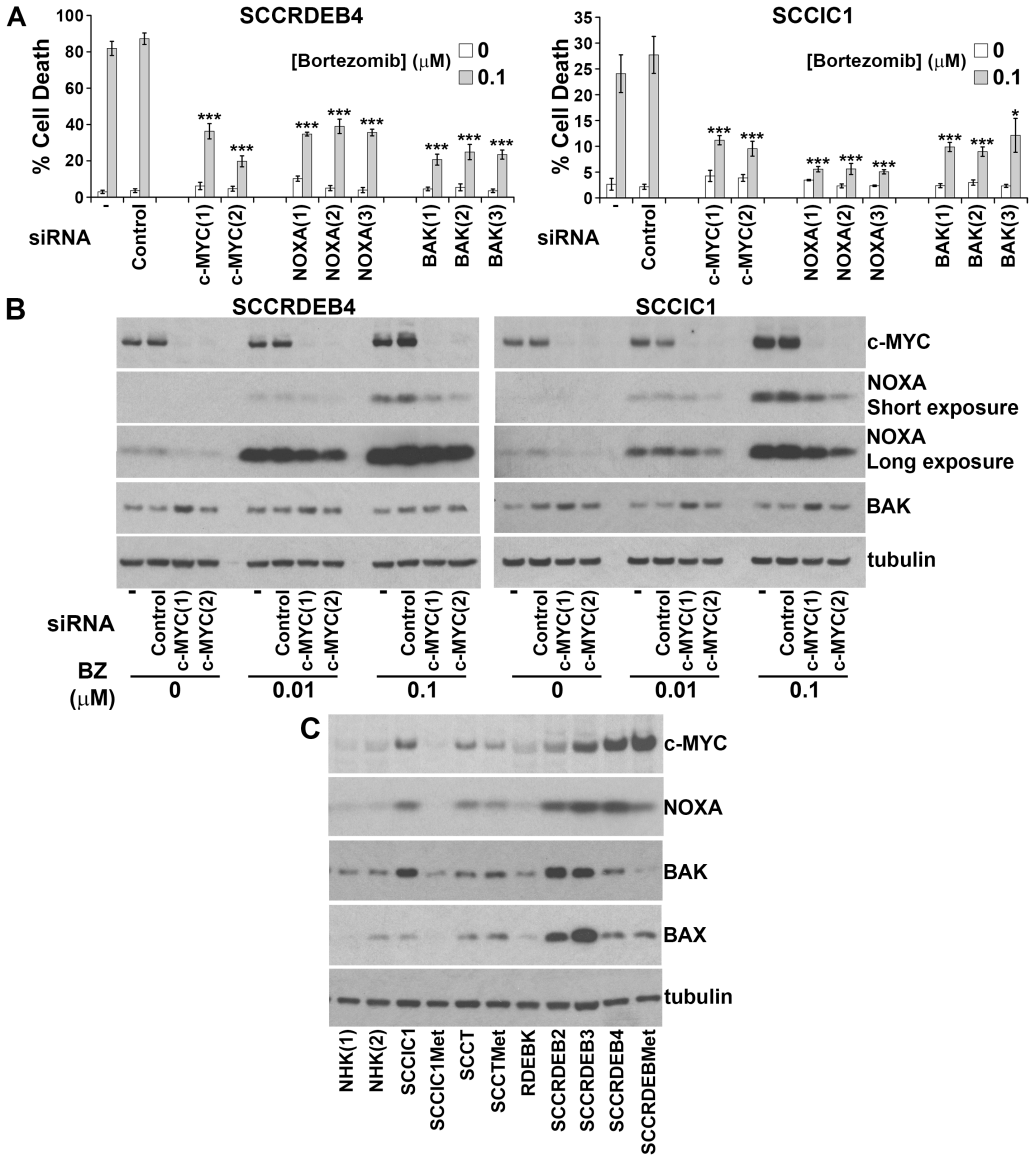
c-MYC-mediated NOXA upregulation is required for BAK-dependent cell death in response to a pulse of bortezomib **(A)** SCCRDEB4 and SCCIC1 cells, in which cell death is sensitive to a pulse of bortezomib, were mock-transfected (-) or transfected with a non-targeting siRNA (Control) or siRNAs targeting c-MYC, NOXA and BAK. siRNAs 1 to 3 are complementary to different sequences within the indicated target. Cells were exposed to an 8-hour pulse of bortezomib and cell death was assessed 24 hours after drug addition by real-time imaging. Values are the mean -/+ SEM of at least 3 independent experiments. Knockdown of c-MYC, NOXA, and BAK attenuated cell death in response to a short exposure to bortezomib (^*^ P < 0.05 and ^***^ P < 0.005 compared with control siRNA transfected cells). **(B)** SCCRDEB4 and SCCIC1 cells were mock-transfected (-) or transfected with non-targeting control siRNA or siRNAs complementary to c-MYC. Cells were treated with carrier or bortezomib (BZ) and were analysed by western blotting 8 hours after drug addition. A cytotoxic concentration of bortezomib (0.1μM) caused c-MYC-dependent accumulation of NOXA. **(C)** The expression of the indicated proteins was analysed by western blotting in the panel of cells used in this study. NHK (1) and (2) are from two different donors. Bortezomib-resistant normal keratinocytes and SCCIC1Met cells expressed low levels of c-MYC and NOXA while bortezomib-resistant SCCRDEBMet cells had low BAK levels.

Clonogenic assays indicated that a 6 to 8-hour treatment with the inhibitors was required for efficient anti-cSCC activity (Supplementary Figure 2). To compare extended and short exposures cells were continuously incubated with inhibitors for 72 hours or treated with an 8-hour pulse of these agents and then maintained in drug-free culture for a further 64 hours. Cell viability (live cell number) and cell death were assayed by real-time imaging. Cell viability was generally reduced at a lower concentration of the agents than required to promote cell death (Figure 1 to 3 and Supplementary Figure 3). This reflects greater inhibitor sensitivity of proliferation.

With continuous exposure to bortezomib there was little selectivity for cSCC compared to untransformed keratinocytes (Figure 1). However, the majority of cSCC cell lines were more sensitive to a pulse of bortezomib than normal keratinocytes. Indeed, a larger window of selectivity for killing sensitive cSCC cells was observed with a pulse of bortezomib than with exposure to carfilzomib or MLN7243 (Figure 1 to 3). The maximum difference in EC50 for killing cSCC cells compared to NHK was 110, 30 and 7.5-fold for bortezomib, carfilzomib and MLN7243 respectively. Furthermore, in half of the cSCC cell lines the EC50 for bortezomib-induced cell death was at least 30-fold lower than for NHK. However, death in SCCRDEBMet and SCCIC1Met cells was insensitive to a short bortezomib exposure.

The general pattern of ixazomib susceptibility in normal keratinocytes and representative cSCC cell lines was similar to that of bortezomib (Supplementary Figure 3). Under the conditions used the reduction in viability and the level of death observed in bortezomib/ixazomib-sensitive SCCRDEB4 cells with a short exposure to low concentrations of ixazomib was less than that with a pulse of bortezomib (Supplementary Figure 3 and Figure 1). This may be related to the faster dissociation rate of ixazomib as the specificity of bortezomib and ixazomib for the proteolytic activities of the proteasome are very similar (Figure 1D and Supplementary Figure 3D) [18-21].

Carfilzomib was more generally selective for killing cSCC cells compared to normal keratinocytes than the other agents tested (Figure 2). The cSCC selectivity was greater with continuous exposure to carfilzomib than with continuous exposure to bortezomib. Under these conditions the EC50 values for killing cSCC cells were 3 to 14-fold lower than for NHK with carfilzomib and a maximum of 2.5-fold lower with bortezomib (Figure 1 and 2). RDEB cSCC cell lines were particularly susceptible to death induced by continuous treatment with carfilzomib. A pulse of carfilzomib exposure resulted in further increases in cSCC selectivity and all cSCC cell lines were killed at substantially lower concentrations of carfilzomib than normal keratinocytes. With a short carfilzomib exposure the EC50 values for killing cSCC cells were 4.5 to 30-fold lower than for NHK.

The ubiquitin E1 inhibitor MLN7243 selectively killed a subset of cSCC lines (Figure 3). SCCT and SCCRDEBMet cells were the most susceptible to continuous treatment with MLN7243. SCCIC1Met cells were also selectively killed by an extended exposure to this agent. Death in SCCRDEBMet cells displayed the greatest sensitivity to a pulse of MLN7243. There were striking differences between responses to this inhibitor and bortezomib: MLN7243-sensitive SCCRDEBMet and SCCIC1Met cells were the most resistant to bortezomib (Figure 1 and 3). Conversely, SCCIC1 cells were susceptible to a pulse of bortezomib but were highly resistant to ubiquitin E1 inhibition.

These results indicate that a good window of selectivity can be achieved with the inhibitors for the killing of cSCC cells derived from primary and metastatic tumours compared to normal keratinocytes but that there are differences in the pattern of inhibitor sensitivity. Clonogenic assays confirmed variations in inhibitor sensitivity observed in the shorter-term real-time imaging assays (Figure 4).

The proteolytic activities of the proteasome were assayed at the end of an 8-hour treatment with proteasome inhibitors, which corresponds to the length of the pulse used for viability/cytotoxicity assays (Figure 1D and 2D and Supplementary Figure 3D). At low doses carfilzomib is more specific for the chymotrypsin-like activity than bortezomib and ixazomib which could underlie the greater cSCC selectivity of continuous carfilzomib exposure (Figure 1 and 2 and Supplementary Figure 3). Bortezomib and ixazomib had similar effects on the proteolytic activities of the proteasome (Figure 1D and Supplementary Figure 3D). Death resulting from a pulse of bortezomib or ixazomib exposure in the most sensitive cSCC cells occurred at inhibitor concentrations that profoundly inhibit both the chymotrypsin and caspase-like activities while having a relatively modest effect on the trypsin-like activity (Figure 1 and Supplementary Figure 3). Killing of normal keratinocytes and resistant cSCC cells was linked with further inhibition of the trypsin-like activity at high inhibitor concentrations. Death in cSCC cells exposed to a pulse of carfilzomib was associated with strong inhibition of the chymotrypsin-like activity and moderate inhibition of both the caspase and trypsin-like activities (Figure 2). Carfilzomib-dependent death in normal keratinocytes was linked with progressive inhibition of the residual activities. A pulse of proteasome inhibitor treatment that specifically blocked the chymotrypsin-like activity of the proteasome caused little or no death in cSCC cell lines. This is consistent with observations in myeloma and breast cancer cells [16, 21]. These results indicate that the level of cSCC selectivity of a pulse of proteasome inhibitor treatment is influenced by the differential dependency for survival in sensitive tumour cells and normal keratinocytes on the proteolytic activities of the proteasome. Differences in the profile of inhibition of the proteolytic activities of the proteasome could contribute to the observed variations in cSCC susceptibility to bortezomib/ixazomib and carfilzomib.

### c-MYC, NOXA and BAK participate in killing cSCC cells in response to a pulse of treatment with proteasome inhibitors

**Figure 6:**
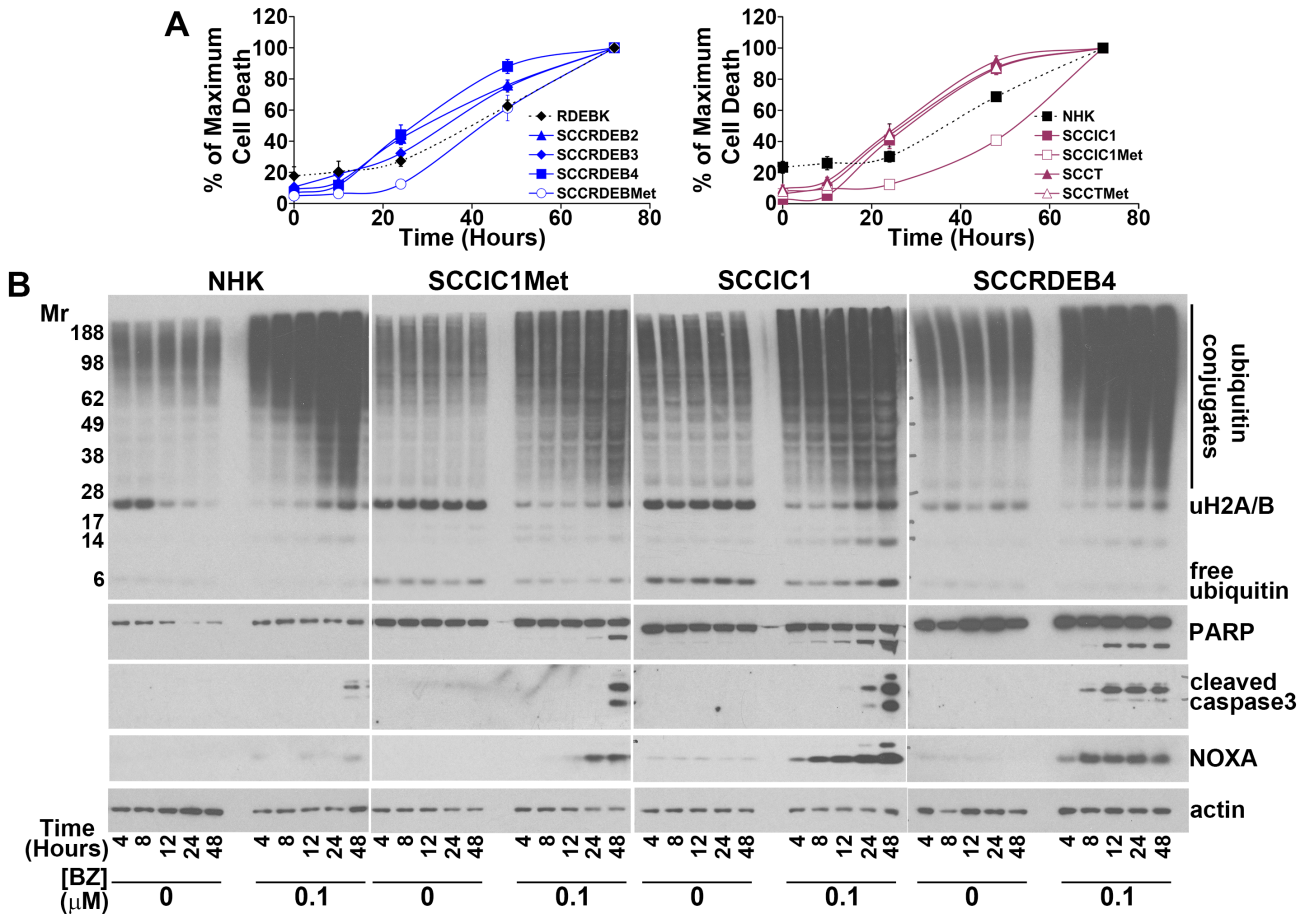
Early induction of NOXA is associated with rapid cell death in cells sensitive to a pulse of bortezomib **(A)** NHK, RDEBK and cSCC cell lines were continuously treated with 0.1 μM bortezomib. The number of dead cells was determined at the indicated time points by real-time imaging. The results are expressed as a percentage of the maximum number of dead cells for each cell type. Values are the mean -/+ SEM of 3 independent experiments. cSCC cell lines sensitive to a pulse of bortezomib die more rapidly than bortezomib-resistant: normal keratinocytes, SCCRDEBMet cells and SCCIC1Met cells. **(B)** cSCC cell lines resistant to a pulse of bortezomib (NHK and SCCIC1Met) and lines sensitive to a short treatment with bortezomib (SCCIC1 and SCCRDEB4) were continuously incubated with carrier (DMSO) or 0.1 μM bortezomib (BZ) for up to 48 hours. The level of ubiquitin and ubiquitin conjugates and the expression of the indicated proteins was analysed by western blotting. Bortezomib caused the accumulation of high molecular weight ubiquitin conjugates in all cells irrespective of their bortezomib-susceptibility. NOXA induction and PARP and caspase 3 cleavage occurred at earlier time points in bortezomib-sensitive cells confirming more rapid apoptosis induction.

We next aimed to identify mechanisms involved in determining sensitivity/resistance to a pulse of treatment with proteasome inhibitors. Basal levels of bulk high molecular weight ubiquitin conjugates were elevated in three of the eight cSCC cell lines (Supplementary Figure 4A). There was a general increase in cSCC cells compared to normal keratinocytes of free monomeric ubiquitin, proteasome subunits and Ser^51^ phosphorylated eiF2ɑ (Supplementary Figure 4). Increased P-eiF2ɑ is consistent with elevated proteotoxic stress in cSCC cells [45]. These changes may reflect a greater demand for proteasomal degradation in the tumour cells compared to normal keratinocytes which could contribute to the greater sensitivity of cSCC cells to proteasome inhibition. However, there was no clear association between these alterations and the resistance of SCCRDEBMet and SCCIC1Met cells to a pulse of bortezomib treatment. Resistance in these cells was not due to a failure of bortezomib to inhibit the proteasome (Figure 1D). Bortezomib-dependent accumulation of bulk high molecular weight ubiquitin conjugates and the return to baseline levels of conjugates after inhibitor removal were indistinguishable in bortezomib-sensitive and resistant cells (Figure 6 and Supplementary Figure 5). Proteasome inhibition reduces the level of mono-ubiquitinated histone H2A/H2B due to depletion of the pool of free ubiquitin [46, 47]. Ubiquitination of these histones is involved in regulation of transcription and DNA damage responses [48]. The pattern of loss and recovery of histone H2A/H2B ubiquitination was also indistinguishable in bortezomib-resistant and sensitive cells (Figure 6 and Supplementary Figure 5). Histone H2A/H2B ubiquitination was recovered even with continuous exposure to bortezomib. There are compensatory mechanisms which act to restore free ubiquitin levels upon proteasome inhibition [49, 50].

To identify mediators of bortezomib sensitivity we explored the effect of knockdown of 40 candidate regulators on the response to bortezomib (data not shown). In bortezomib-sensitive SCCRDEB4 and SCCIC1 cells siRNA-mediated depletion of c-MYC and the pro-apoptotic proteins NOXA and BAK was found to attenuate cell death in response to a pulse of bortezomib (Figure 5A). BAK is an apoptosis effector that regulates mitochondrial membrane permeability [51]. siRNAs targeting other apoptosis effectors BAX and BOK did not diminish bortezomib-dependent cell death in SCCRDEB4 cells (Supplementary Figure 6). A particular dependence on BAK is consistent with previous observations in mesothelioma [52]. c-MYC and NOXA are both degraded by the proteasome [53-55]. NOXA is a transcriptional target of c-MYC and NOXA activates BAK by binding to the BAK repressor MCL-1 [56, 57]. In SCCRDEB4 and SCCIC1 cells a cytotoxic concentration of bortezomib increased c-MYC and NOXA protein expression within 8 hours of drug treatment (Figure 5B). Depletion of c-MYC reduced the level of NOXA accumulated by bortezomib indicating that c-MYC participates in NOXA upregulation. This is consistent with a role of c-MYC/NOXA/BAK acting in a common pathway to mediate cell death in response to a pulse of bortezomib.

Basal expression of c-MYC and NOXA was low in bortezomib-resistant normal keratinocytes and SCCIC1Met cells and BAK levels were low in the bortezomib-resistant SCCRDEBMet cell line (Figure 5C). Low expression of one or more of these proteins could contribute to resistance to a pulse of bortezomib treatment. c-MYC expression was increased in all cSCC cell lines with elevated NOXA and knockdown of c-MYC reduced basal NOXA expression (Figure 5B and 5C). This indicates that c-MYC is required to maintain elevated basal levels of NOXA. cSCC cell lines sensitive to a pulse of bortezomib died faster than resistant cells when exposed to continuous bortezomib treatment (Figure 6A and 6B). This was associated with the accumulation of NOXA at earlier time points in cells sensitive to a pulse of bortezomib (Figure 6B). These results suggest that cells can be sensitive to a pulse of bortezomib because they die rapidly in response to bortezomib treatment. High expression of c-MYC/NOXA may confer sensitivity to a short bortezomib exposure by priming cSCC cells for fast bortezomib-mediated cell death.

c-MYC/NOXA/BAK also contribute to promoting rapid cell death in response to a pulse of carfilzomib treatment in bortezomib-sensitive cSCC cells (Supplementary Figure 7). In carfilzomib-sensitive but bortezomib-resistant SCCRDEBMet cells death in response to a pulse of carfilzomib was not affected by targeting c-MYC, NOXA or BAK (Supplementary Figure 7A). This indicates that short-term treatment with carfilzomib can kill these cells through another mechanism. The engagement of an alternative mechanism of cell death, possibly due to differences in the pattern of proteasome inhibition, could account for the greater sensitivity of SCCRDEBMet cells to a pulse of carfilzomib.

### UBA1 protein levels influence sensitivity to MLN7243

**Figure 7:**
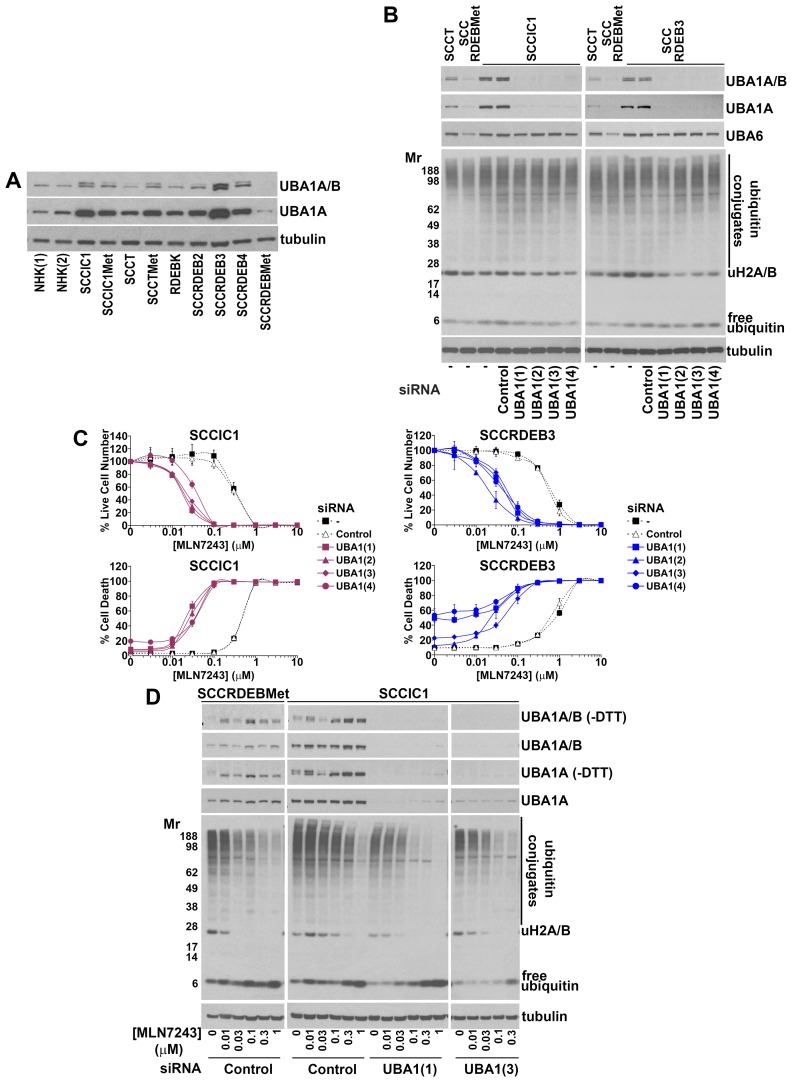
Low UBA1 protein expression is a determinant of sensitivity to MLN7243 **(A)** The expression of UBA1 in the panel of cells used in this study was analysed by western blotting. This was carried out using a primary antibody that detects both major UBA1 isoforms: UBA1A (upper band) and UBA1B (lower band) and an antibody that is specific for UBA1A. UBA1A and B expression was lowest in MLN7243-sensitive SCCRDEBMet and SCCT cells while UBA1A expression was high in MLN7243-resistant SCCRDEB3 and SCCIC1 cells. **(B)** MLN7243-resistant SCCIC1 and SCCRDEB3 cells were mock-transfected (-) or transfected with the indicated siRNAs. UBA1 siRNAs 1 to 4 are complementary to different UBA1 sequences. Ubiquitin E1 expression and the pattern of ubiquitination were analysed by western blotting 72 hours after transfection. The UBA1 siRNAs efficiently depleted UBA1 while having no effect on the level of UBA6 or bulk high molecular weight ubiquitin conjugates. **(C)** MLN7243-resistant SCCIC1 and SCCRDEB3 cells were transfected with siRNAs targeting UBA1. Cell viability (live cell number) expressed as a percentage of carrier alone for each siRNA and cell death were analysed by real-time imaging 72 hours after the initiation of continuous incubation with MLN7243. Values are the mean -/+ SEM of 3 experiments. Knockdown of UBA1 dramatically increased sensitivity to MLN7243. **(D)** MLN7243-sensitive SCCRDEBMet cells and MLN7243-resistant SCCIC1 cells were transfected with the indicated siRNAs. Samples were analysed by western blotting for UBA1 and ubiquitin 12 hours after initiating continuous incubation with MLN7243. More slowly migrating UBA1-ubiquitin thioesters were preserved by running samples in the absence of reducing agent (-DTT). MLN7243 decreased UBA1 thioesters. Cells with low UBA1 expression were more sensitive to MLN7243-induced decreases in high molecular weight ubiquitin conjugates and ubiquitinated histone H2A/H2B.

To identify determinants of sensitivity to MLN7243 the protein expression of the ubiquitin E1 UBA1 was compared in the panel of cells used in this study (Figure 7A). Two major isoforms of UBA1 are generated from a common mRNA as a result of alternative start codon usage [58-60]. UBA1A is localised in the nucleus and the cytoplasm. UBA1B lacks the 40 N-terminal amino acid residues of UBA1A and is predominantly cytoplasmic. In cSCC cells there was an inverse association between MLN7243 sensitivity and UBA1 expression. UBA1A and B expression was low in SCCRDEBMet and SCCT cells which were the most MLN7243-sensitive. SCCRDEBMet cells had the lowest levels of both UBA1 isoforms and death in these cells was the most sensitive to a pulse of MLN7243 (Figure 3). Conversely, UBA1A expression was high in the SCCIC1 and SCCRDEB3 lines which were the most MLN7243-resistant. Knockdown of UBA1A and B in these MLN7243-resistant lines dramatically increased sensitivity to the effects of the E1 inhibitor on cell viability and death (Figure 7B and 7C). The level of UBA1 expression attained following siRNA-mediated knockdown in these resistant lines and the resulting susceptibility to MLN7243 were comparable to those in MLN7243-sensitive SCCRDEBMet cells (Figure 3 and 7B and 7C). Consistent with inhibition of ubiquitin E1s, MLN7243 decreased the level of DTT-sensitive UBA1 species (UBA1-ubiquitin thioesters) along with high molecular weight ubiquitin conjugates and ubiquitinated histones H2A/H2B while the level of free monomeric ubiquitin was increased (Figure 7D). These effects occurred at lower MLN7243 concentrations in sensitive SCCRDEBMet cells than in resistant SCCIC1 cells. UBA1 knockdown in SCCIC1 cells reduced the concentration of MLN7243 required to attenuate ubiquitination. In all circumstances cell death occurred at doses of MLN7243 that diminished ubiquitin conjugates. These results indicate that UBA1 levels are a major determinant of the sensitivity of cSCC cells to MLN7243 due to their influence on the concentration of this agent required to inhibit ubiquitination. In contrast, UBA6 protein expression was relatively uniform across the panel of cells (Supplementary Figure 8A). Knockdown of UBA6 in MLN7243-resistant SCCIC1 and SCCRDEB3 cells did not alter their sensitivity to the E1 inhibitor (Supplementary Figure 8B and 8C). This indicates that UBA6 protein levels are not a determinant of MLN7243 sensitivity in these cell lines.

Knockdown of UBA1 did not greatly alter the pattern of bulk high molecular weight ubiquitin conjugates in cSCC cells, even in the SCCRDEBMet line which expresses the lowest endogenous levels of the E1 (Figure 7 and Supplementary Figure 9). In addition, knockdown of UBA6 alone and in combination with UBA1 did not alter high molecular weight ubiquitination in SCCRDEBMet cells (Supplementary Figure 9B). This suggests that ubiquitin E1s are not normally rate-limiting for the formation of these ubiquitin conjugates in the cSCC cell lines tested and that in these cells E1 activity needs to be strongly inhibited in order for ubiquitination to be attenuated. Levels of ubiquitinated histone H2A/H2B were reduced by knockdown of UBA1 (Figure 7B and 7D and Supplementary Figure 9). A decrease in ubiquitinated histone H2A/H2B could provide a sensitive readout of UBA1 suppression.

## DISCUSSION

We compared the effects in normal keratinocytes and cSCC cell lines of a long and short treatment with proteasome inhibitors and the ubiquitin E1 inhibitor MLN7243. We show that a pulse of proteasome inhibitor exposure results in greater selectivity for killing cSCC cells than a more extended exposure. In sensitive cSCC cell lines a pulse of bortezomib treatment gives the highest level of selectivity compared to normal keratinocytes but some cSCC cell lines are bortezomib-resistant. These bortezomib-resistant tumour cells can be sensitive to MLN7243. Carfilzomib is more generally cSCC selective than bortezomib or MLN7243. We provide evidence that c-MYC, NOXA and BAK are determinants of susceptibility to a pulse of proteasome inhibition and that low UBA1 expression confers susceptibility to MLN7243.

This study indicates that the cSCC selectivity of a pulse of proteasome inhibitor exposure is related to a differential dependency for survival of sensitive cSCC cells and normal keratinocytes on the proteolytic activities of the proteasome. For example, killing of the most bortezomib-sensitive cSCC cell lines by a short treatment with bortezomib occurs at inhibitor concentrations that markedly attenuate the chymotrypsin and caspase-like activities but only modestly inhibit the trypsin-like activity. In normal keratinocytes death is associated with inhibition of all three activities at high bortezomib concentrations. This suggests that the window of selectivity for cSCC cells compared to normal keratinocytes can be determined by the specificity of inhibitors for the activities of the proteasome. For instance, a small molecule which inhibits the chymotrypsin and caspase-like activities without substantially inhibiting the trypsin-like activity may show even greater selectivity for sensitive cSCC cells than bortezomib. These results are consistent with the previous finding that inhibition of different individual proteolytic activities and combinations of proteolytic activities of the proteasome results in distinct degrees of interference with protein degradation [15, 16]. The extent to which the proteolytic activities are inhibited could consequently influence which cellular pathways are affected and the strength of their modulation.

We observed that concentrations of bortezomib and carfilzomib that kill cSCC cell lines have divergent effects on the proteolytic activities of the proteasome. Variations in the extent to which cellular pathways are affected due to these differences in proteasome inhibition may underlie the greater cSCC selectivity of continuous exposure to carfilzomib and the ability of a pulse of carfilzomib to kill cSCC cells resistant to a short treatment with bortezomib. Consistent with this, our study indicates that a pulse of carfilzomib can induce cell death by different mechanisms in bortezomib-resistant and bortezomib-sensitive cells. Differences in the mechanisms through which distinct proteasome inhibitors kill cells have been observed previously [61].

c-MYC, NOXA and BAK promote cell death in response to an extended exposure to bortezomib in a number of cancer types [43, 52, 54, 55, 57, 62, 63]. This study confirms that this pathway can play an important role in mediating cell death induced by a pulse of bortezomib. In addition, we observed that this pathway can also participate in killing bortezomib-sensitive cSCC lines by a pulse of carfilzomib. c-MYC and NOXA levels are low in normal keratinocytes which provides a mechanism for tumour selectivity. Furthermore, cSCC cell lines resistant to a pulse of bortezomib have low basal levels of c-MYC and NOXA or BAK. Low basal expression of these proteins is consequently a potential marker for bortezomib resistance in cSCCs. In support of this, a decrease in c-MYC and NOXA expression is involved in the acquired resistance of mesothelioma cells to bortezomib [52]. Encouragingly, c-MYC protein expression is upregulated in 75 to 85% of cSCCs and BAK protein is expressed in 95% of cSCCs [64-67]. Our results indicate that cells are sensitive to a pulse of bortezomib because they undergo more rapid bortezomib-induced death than resistant cells. Early c-MYC-dependent accumulation of the BAK activator NOXA contributes to promoting quicker cell death in bortezomib-sensitive cells. Both c-MYC and NOXA are degraded by the proteasome and c-MYC promotes *NOXA* transcription. Upregulation of basal NOXA mRNA expression by elevated c-MYC could contribute to fast protein accumulation upon inhibition of the proteasomal degradation of NOXA. Proteasome inhibition also increases c-MYC transcriptional activity towards the *NOXA* gene [52, 57, 68].

The pattern of sensitivity of cSCC cells to ubiquitin E1 inhibition is different from proteasome inhibitors. The two cSCC cell lines most resistant to a pulse of bortezomib are among the most sensitive to MLN7243-induced cell death. We observed that low expression of both UBA1A and B isoforms is associated with MLN7243 sensitivity and high expression of UBA1A with MLN7243 resistance. Furthermore, knockdown of UBA1A and B confers MLN7243 sensitivity to resistant cSCC cells. MLN7243-induced cell death is associated with a reduction in the level of ubiquitin conjugates. A lower concentration of MLN7243 is required to diminish ubiquitination in cSCC cells with low UBA1 expression. UBA1 protein levels may thus provide a marker for tumour sensitivity to MLN7243. It would be of great interest to determine the mechanisms responsible for the observed differences in UBA1A and B expression. A better understanding of how levels of UBA1 isoforms are regulated could lead to the development of therapeutic interventions that modulate their expression. This may provide a means to enhance tumour sensitivity or increase the resistance of normal cells to MLN7243. UBA1 and UBA6 are both inhibited by MLN7243. While our study supports a pre-eminent role of UBA1 in determining the sensitivity of cSCC cells to MLN7243 under some circumstances suppression of UBA6 could contribute to the anti-tumour activity of this inhibitor. UBA6 can play a non-redundant role in maintaining cell viability [10, 69].

A high degree of E1 suppression is required to reduce bulk high molecular weight ubiquitin conjugates in the cSCC cell lines examined. This indicates that the ubiquitin E1s are not normally rate-limiting for these ubiquitination events. This is consistent with previous studies [38]. UBA1 is a highly active enzyme and it is able to charge excess amounts of E2s with ubiquitin [70]. There are examples of cancer-derived cells, including acute and chronic myeloid leukaemia cell lines where UBA1 is closer to rate-limiting for ubiquitination [37]. These cancers may be highly sensitive to MLN7243.

This study indicates that there is therapeutic potential for proteasome and UBA1 inhibition for cSCC. Future work could initially be aimed at developing direct delivery of inhibitors to tumours. This would overcome limitations of systemic delivery and allow optimal exposure of cSCCs. Effective directly-delivered therapy would be of benefit to cSCC patients and it would inform systemic therapy for treating cSCC and other solid tumours.

## MATERIALS AND METHODS

### Cell culture

Normal keratinocytes (NHK and RDEBK) and cSCC cell lines were isolated and maintained as described [71, 72]. Cells were routinely grown at 37°C and 5% CO_2_ in a humidified atmosphere in keratinocyte medium containing 10% serum and growth factors [72]. Normal keratinocytes were grown in the presence of a mitotically inactivated 3T3 fibroblast feeder layer. RDEBK were expanded using the ROCK inhibitor Y-27632 (1254: Tocris Bioscience, Bristol, UK) which was removed 48 hours prior to the initiation of experiments [73]. cSCC cell lines were cultured in the absences of feeder cells. SCCRDEBMet (SCCRDEB70) and RDEBK cells were a gift from Dr Andrew P. South (Thomas Jefferson University). The tissue from which these cells were derived was provided by Jemima E. Mellerio (King’s College London) and Julio C. Salas-Alanís (DEBRA Mexico). SCCT (MET1) and SCCTMet (MET4) cell lines were described previously [74]. SCCIC1/SCCIC1Met and SCCT/SCCTMet cell lines are derived from paired primary tumours and their metastases. For experiments, cells were plated in the absence of feeders in keratinocyte medium containing 10% serum and growth factors with no added EGF.

### Inhibitor treatment

Inhibitors were dissolved in DMSO (20 to 50 mM stocks). Unless otherwise indicated cells were treated with inhibitors 16 to 24 hours after plating. Compounds used in this study were: bortezomib (B-1408: LC Laboratories Woburn, MA, USA), carfilzomib (S2853: Selleckchem, Houston, TX, USA), ixazomib (S2180: Selleckchem) and MLN7243 (CT-M7243: Chemietek, Indianapolis, IN, USA).

### siRNA transfection

Dharmacon ON-TARGETplus modified siRNAs (Thermo Fisher Scientific, Waltham, MA, USA) were used in this work and are listed in Supplementary Figure 10. Reverse transfection with synthetic siRNA duplexes (10 nM) was carried out using Invitrogen Lipofectamine RNAiMAX transfection reagent (13778150: Thermo Fisher Scientific) according to the manufacturer’s instructions. Inhibitors were added 48 hours after transfection to ensure target knockdown before initiating drug treatment.

### Cell viability assays

Cells were seeded into 96 well plates and live cell number and cell death were analysed according to the manufacturers’ instructions with an IncuCyte ZOOM real-time imager (Essen BioScience Ltd, Welwyn Garden City, UK) using the CellTox Green Cytotoxicity Assay (G8731: Promega, Southampton, UK). Relative EC50 values were determined using GraphPad Prism (GraphPad Software Inc, La Jolla, CA, USA). For clonogenic assays cells were seeded into six well plates. Inhibitors were added for the indicated times and then cells were maintained in drug-free medium for up to 2 weeks to allow colony formation. Colonies were fixed in 10% methanol, 10% acetic acid and stained with crystal violet.

### Statistical analysis

The statistical significance of differences between two groups (control and targeting siRNA) was determined using a one tailed T-test.

### Proteasome activity measurements

The three proteolytic activities of the proteasome were measured in parallel in cells seeded in 96 well plates using the Cell-Based Proteasome-Glo Assay (G1180: Promega). Medium from wells containing no cells was used as a blank.

### Western blotting

The primary antibodies used are listed in Supplementary Figure 10. Cell extracts were prepared by direct lysis into SDS electrophoresis sample buffer: 100 mM Tris pH 6.8, 4% SDS, 20% glycerol, 20 mM EDTA, 0.014% bromophenol blue. Western blotting was carried out as described previously [75]. Membranes probed for ubiquitin were boiled for 30 minutes in de-ionized water prior to blocking to increase epitope exposure.

## SUPPLEMENTARY MATERIALS FIGURES



## Abbreviations

(BZ): Bortezomib
(cSCC): cutaneous squamous cell carcinoma
(uH2A/B): mono-ubiquitinated histone H2A/H2B
(NHK): normal human keratinocytes
(RDEB): recessive dystrophic epidermolysis bullosa
(RDEBK): recessive dystrophic epidermolysis bullosa keratinocytes
(SCCIC): squamous cell carcinoma immunocompetent
(SCCRDEB): squamous cell carcinoma recessive dystrophic epidermolysis bullosa
(SCCT): squamous cell carcinoma transplant
(UBL): ubiquitin-like
