# Supplementary material for: Preclinical comparison of proteasome and ubiquitin E1 enzyme inhibitors in cutaneous squamous cell carcinoma: the identification of mechanisms of differential sensitivity
Source: Oncotarget. 2018 Apr 17;9(29):20265–81. doi: 10.18632/oncotarget.24750 (PMC5945540; doi:10.18632/oncotarget.24750)
Supplement: Supplementary file 1 [file oncotarget-09-20265-s001.pdf]

Preclinical comparison of proteasome and ubiquitin E1 enzyme inhibitors in cutaneous squamous cell carcinoma: the identification of mechanisms of differential sensitivity

SUPPLEMENTARY MATERIALS

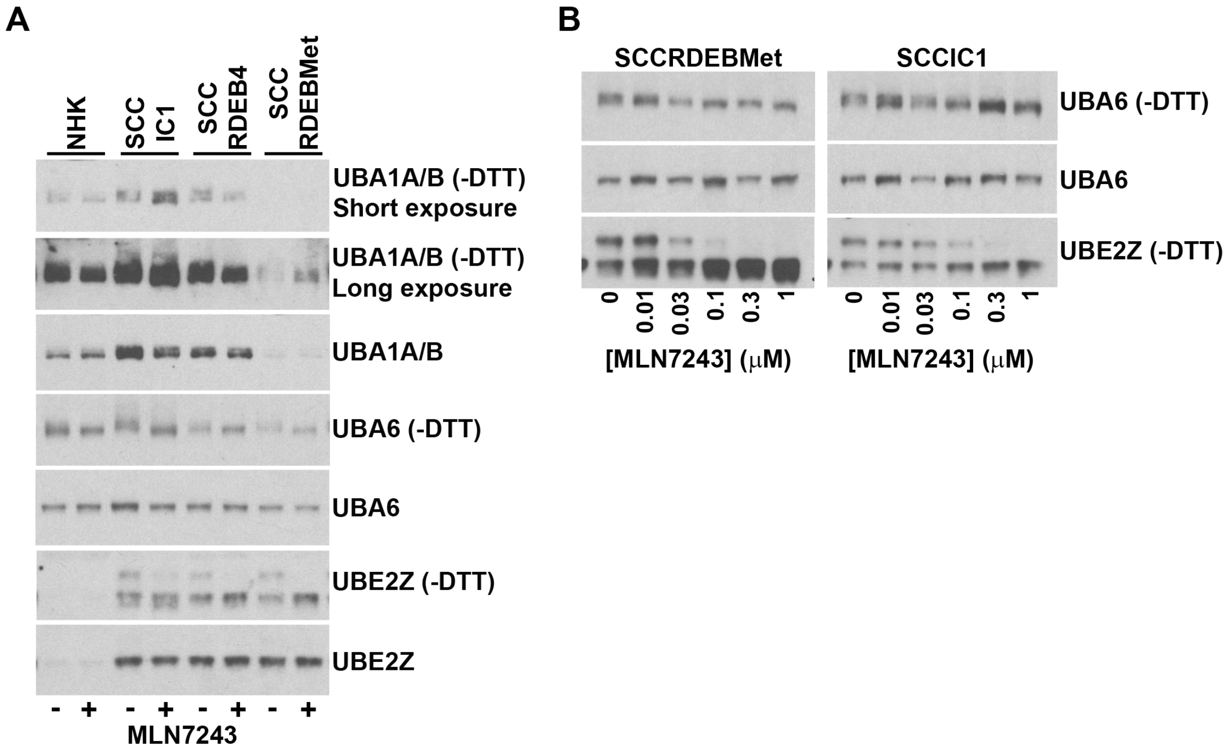

Supplementary Figure 1: MLN7243 inhibits both UBA1 and UBA6. UBA1 and UBA6 form reducing agent-sensitive thioesters with ubiquitin. UBA6 also forms a thioester with the UBL protein FAT10. UBA6 but not UBA1 can transfer ubiquitin and FAT10 to the E2 enzyme UBE2Z/USE1 to form a UBE2Z thioester. **(A)** The indicated cells were treated with carrier (-) or 0.1μM MLN7243 (+) for 12 hours. Samples were analysed by western blotting. A primary antibody that recognises both isoforms of UBA1 was used. Slower mobility thioesters of UBA1, UBA6 and UBE2Z were detected in the absence of reducing agent (-DTT). The molecular weight of ubiquitin and FAT10 are 8 and 18 kDa respectively. The migration of the detected reducing agent-sensitive form of UBE2Z indicated that it corresponded to a ubiquitin-UBE2Z thioester. MLN7243 inhibited UBA1, UBA6 and UBE2Z-thioester formation. **(B)** SCCRDEBMet and SCCIC1 cell lines were treated with MLN7243 for 12 hours and analysed by western blotting. MLN7243 inhibited UBA6 and UBE2Z thioester formation in a dose-dependent manner.

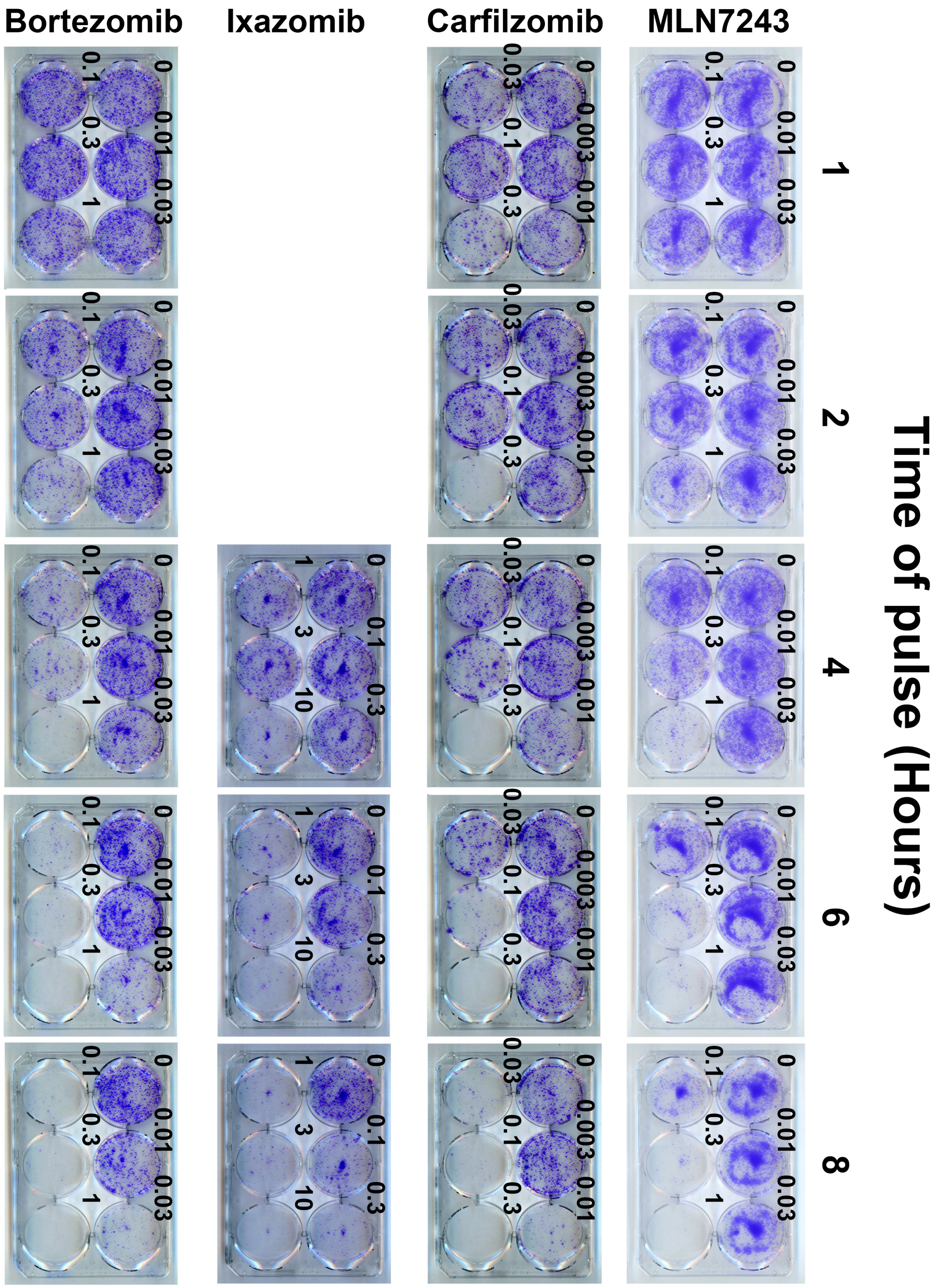

Supplementary Figure 2: The effect of the time of inhibitor exposure on colony formation. SCCRDEB4 cells were treated with inhibitors for 1 to 8 hours. Cells were then placed into drug-free medium to allow colony formation. A reduction in colony formation could be observed with a 2 to 4-hour pulse of inhibitor treatment while longer exposure times resulted in sensitivity to lower concentrations of the agents.

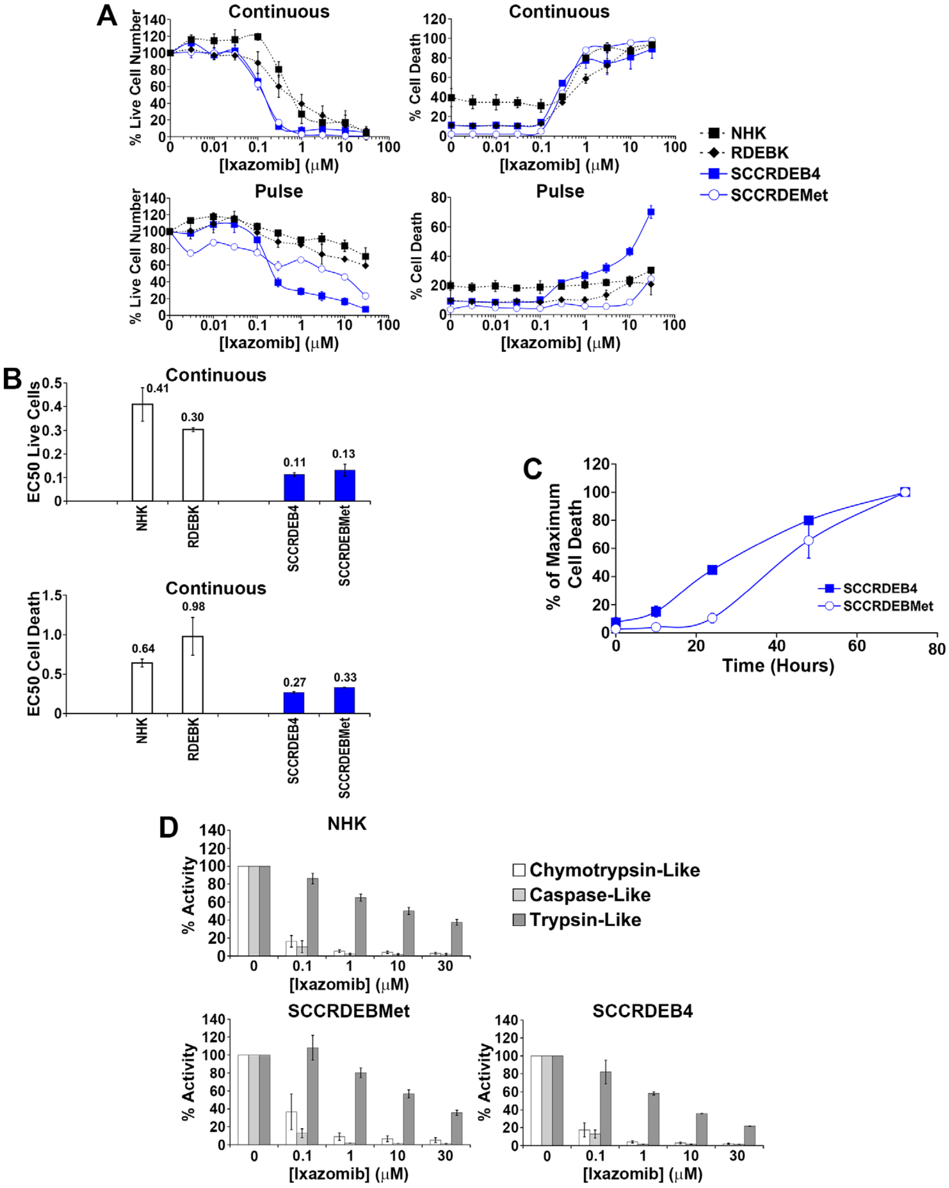

Supplementary Figure 3: The pattern of ixazomib sensitivity in normal keratinocytes and representative cSCC cell lines is similar to that of bortezomib. (A and B) NHK, RDEBK and SCCRDEBMet cells (resistant to a pulse of bortezomib) and SCCRDEB4 cells (sensitive to a pulse of bortezomib) were continuously incubated with the proteasome inhibitor ixazomib or treated with an 8-hour ixazomib pulse. Cell viability (live cell number), expressed as a percentage of carrier alone and the percentage of dead cells were determined by real-time imaging 72 hours after drug addition. Values are the mean -/+ SEM of 3 independent experiments. (A) Ixazomib dose-response curves. (B) Relative EC50 values (μM) for reducing cell viability (live cell number) and for promoting cell death. There was little selectivity with continuous exposure to ixazomib but death in SCCRDEB4 cells was the most sensitive to a pulse of ixazomib. **(C)** Cells were continuously treated with 1μM ixazomib. The number of dead cells was determined at the indicated time points by real-time imaging. The results are expressed as a percentage of the maximum number of dead cells for both cell lines. Values are the mean -/+ SEM of 3 independent experiments. As with bortezomib SCCRDEB4 cells died more rapidly than SCCRDEBMet cells. **(D)** The proteolytic activities of the proteasome were assayed 8 hours after the addition of ixazomib. The results were expressed as a percentage of the activity with carrier alone. Values are the mean -/+ range of 2 independent experiments. Increased death of SCCRDEB4 cells resulting from a pulse of ixazomib exposure first occurred at ixazomib concentrations that strongly inhibit both the chymotrypsin and caspase-like activities of the proteasome.

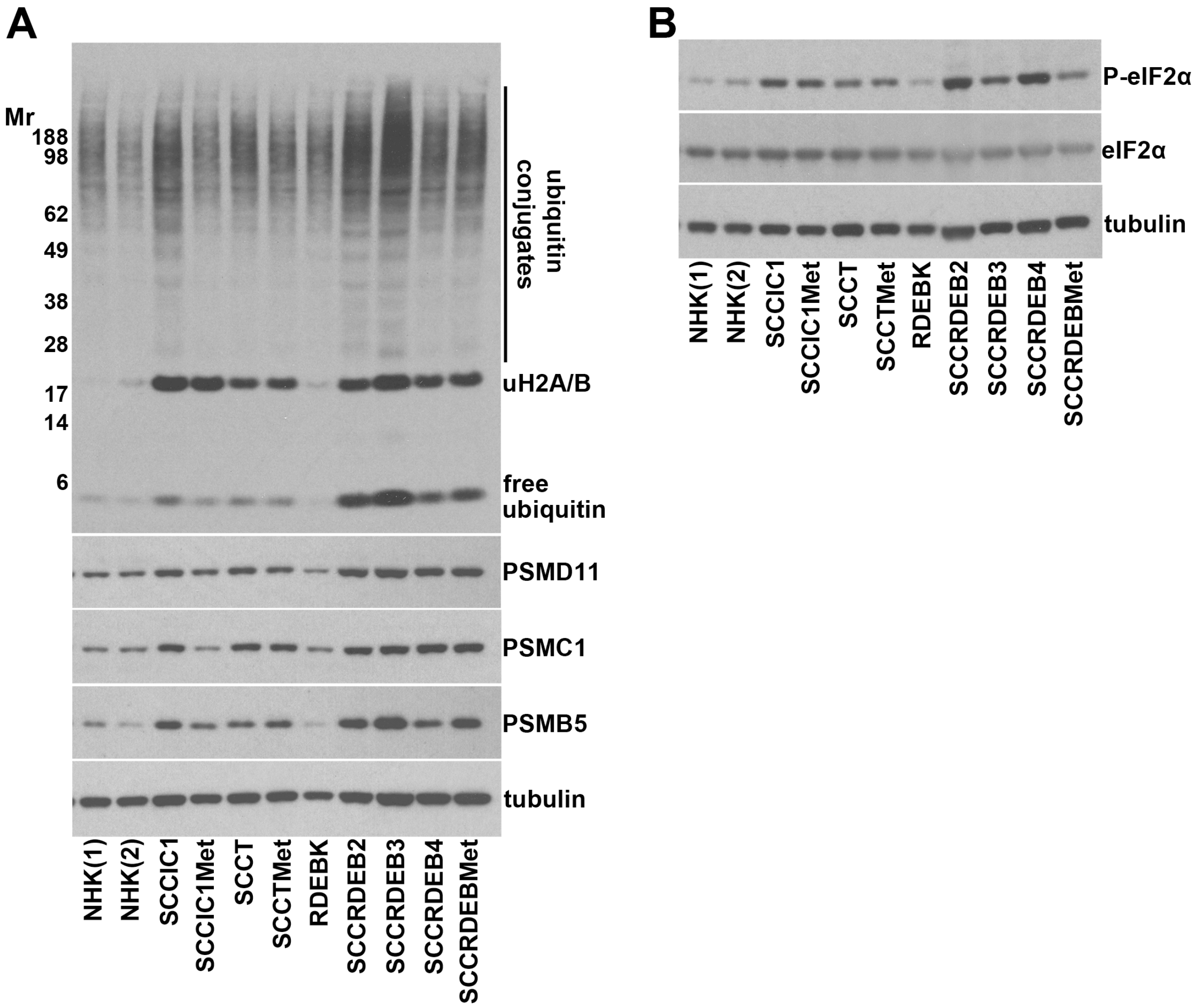

Supplementary Figure 4: Comparison of the: pattern of ubiquitination, expression of proteasome subunits and levels of P-eiF2α in normal keratinocytes and cSCC cell lines. (A and B) The panel of cells used in this study was analysed by western blotting. (A) The level of bulk high molecular weight ubiquitin conjugates was highest in SCCIC1, SCCRDEB2 and SCCRDEB3 cells. The levels of mono-ubiquitinated histone H2A/H2B (uH2A/B) and free ubiquitin were elevated in cSCC cells compared to normal keratinocytes. Expression of the proteasome subunits PSMB5/β5 (20S core), PSMC1/S4/Rpt2 (19S base) and PSMD11/S9/Rpn6 (19S lid) was higher in cSCC cells compared to keratinocytes. (B) Ser^{51} phosphorylation of eiF2α is increased in cSCC cells.

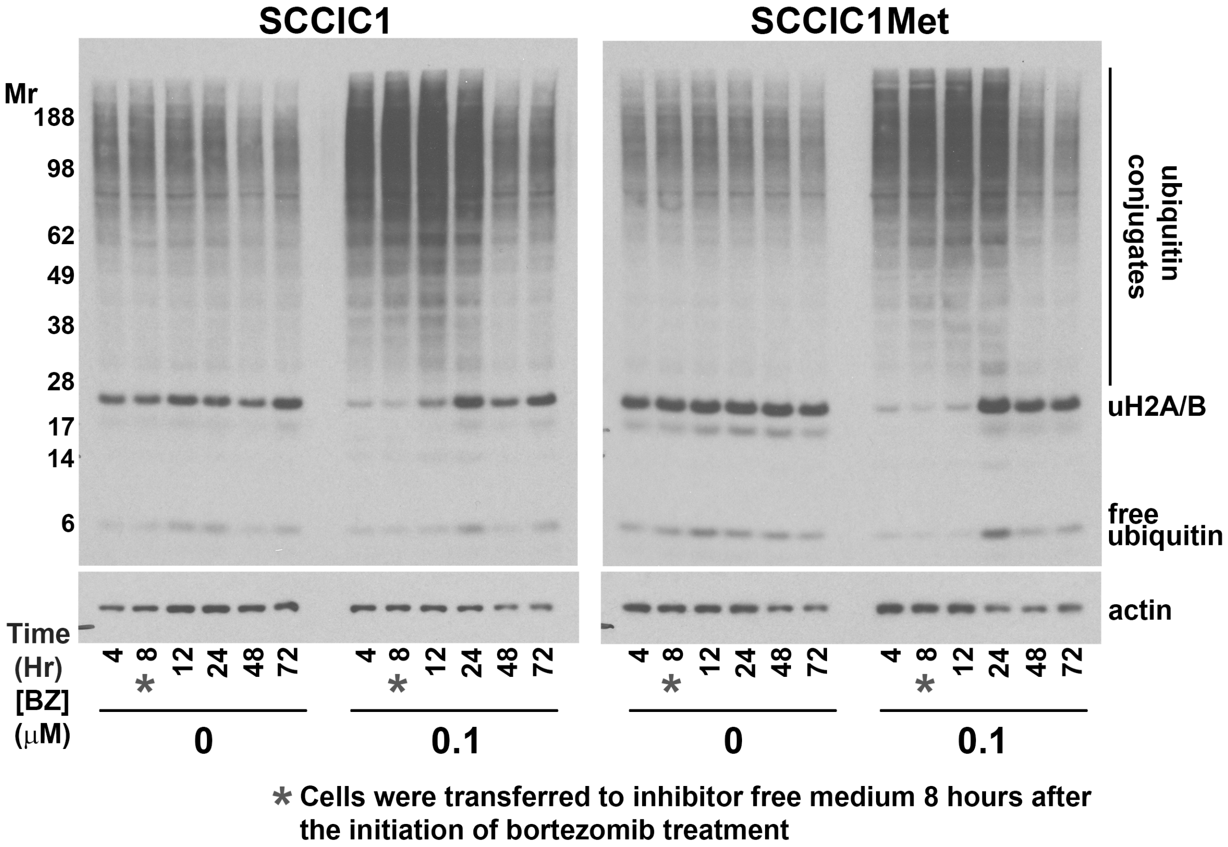

Supplementary Figure 5: A pulse of bortezomib has similar effects on the pattern of ubiquitination in bortezomib-sensitive and bortezomib-resistant cSCC cells. The SCCIC1 cell line which is sensitive to a pulse of bortezomib and the paired metastatic SCCIC1Met cell line which is resistant to a pulse of bortezomib were treated with carrier or 0.1 μM bortezomib (BZ) for 8 hours. The cells were incubated in bortezomib-free medium for the indicated time after drug addition and analysed by western blotting for ubiquitin. The effect on the pattern of ubiquitination was similar in the cell lines: High molecular weight ubiquitin conjugates were accumulated after bortezomib addition and returned to basal after inhibitor removal at similar rates. Ubiquitinated histone H2A/H2B levels were reduced by bortezomib and returned to basal levels with similar kinetics.

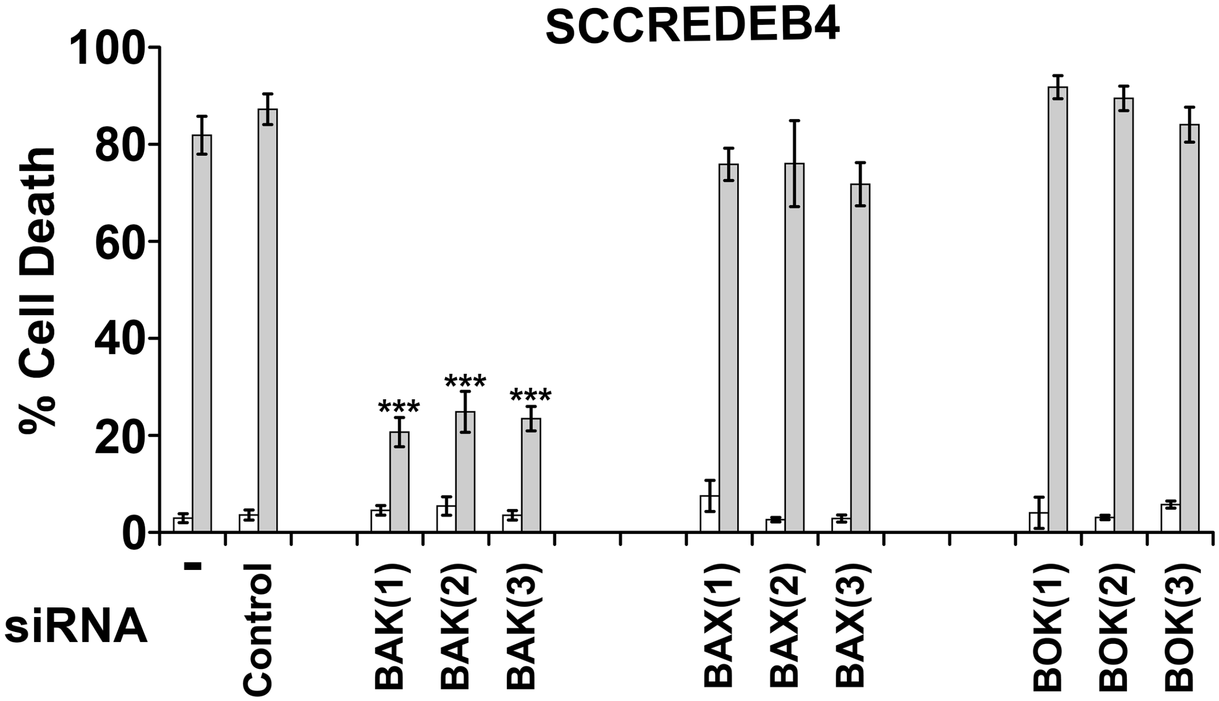

Supplementary Figure 6: BAX and BOK are not rate-limiting for cell death induced by a pulse of bortezomib. SCCRDEB4 cells were mock-transfected (-) or transfected with a non-targeting siRNA (Control) or 3 different siRNAs targeting the apoptosis effectors BAK, BAX and BOK. Cells were treated with bortezomib for 8 hours and then transferred to inhibitor-free medium. The percentage of dead cells was assayed by real-time imaging 24 hours after drug addition. Values are the mean -/+ SEM of at least 3 independent experiments. Only BAK siRNAs attenuated bortezomib-induced cell death (*** $P < 0.005$ compared with control siRNA transfected cells).

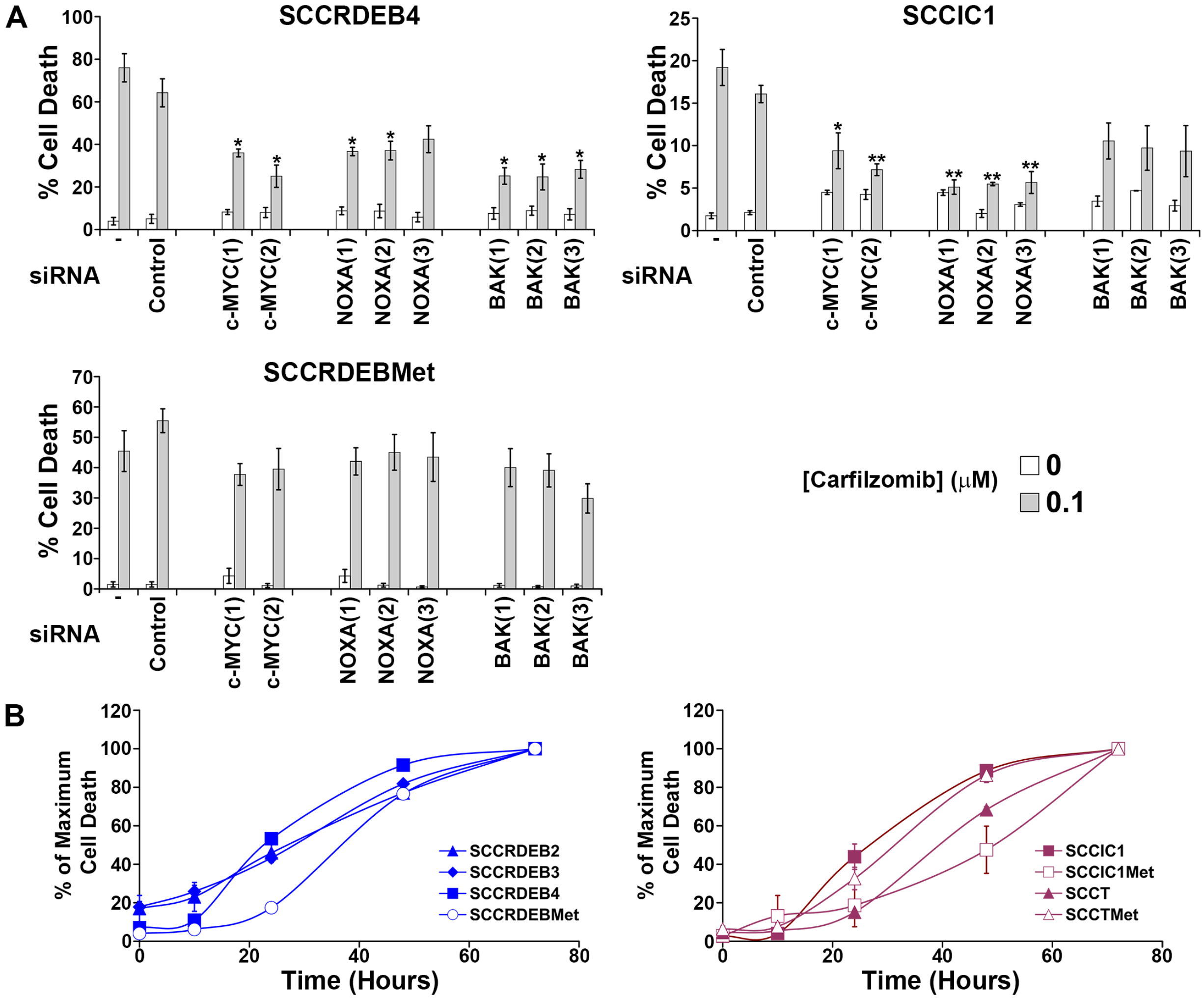

Supplementary Figure 7: Investigation of the role of c-MYC, NOXA and BAK in cSCC death induced by a pulse of carfilzomib. **(A)** cSCC cell lines were mock-transfected (-) or transfected with the indicated siRNAs. Cells were treated with 0.1 μM carfilzomib for 8 hours and then incubated in inhibitor-free medium for a total of 24 (SCCRDEB4 and SCCIC1 cells) or 48 hours (SCCRDEBMet cells) after drug addition. The percentage of dead cells was determined by real-time imaging. Values are the mean -/+ SEM of 3 independent experiments. siRNAs targeting c-MYC, NOXA and BAK reduced cell death in response to carfilzomib in SCCRDEB4 cells which are sensitive to a pulse of bortezomib (* $P < 0.05$ compared with control siRNA transfected cells). siRNAs targeting c-MYC and NOXA attenuated carfilzomib-dependent death in bortezomib-sensitive SCCIC1 cells (* $P < 0.05$, ** $P < 0.01$ compared with control siRNA transfected cells). c-MYC, NOXA and BAK siRNAs had little effect on carfilzomib-induced death in bortezomib-resistant SCCRDEBMet cells. **(B)** Cells were continuously treated with 0.1 μM carfilzomib. The number of dead cells was determined at the indicated time points by real-time imaging. The results are expressed as a percentage of the maximum number of dead cells for each cell line. Values are the mean -/+ SEM of 3 independent experiments. As with 0.1 μM bortezomib (Figure 6) the rate of cell death was slow in SCCRDEBMet and SCCIC1Met cells.

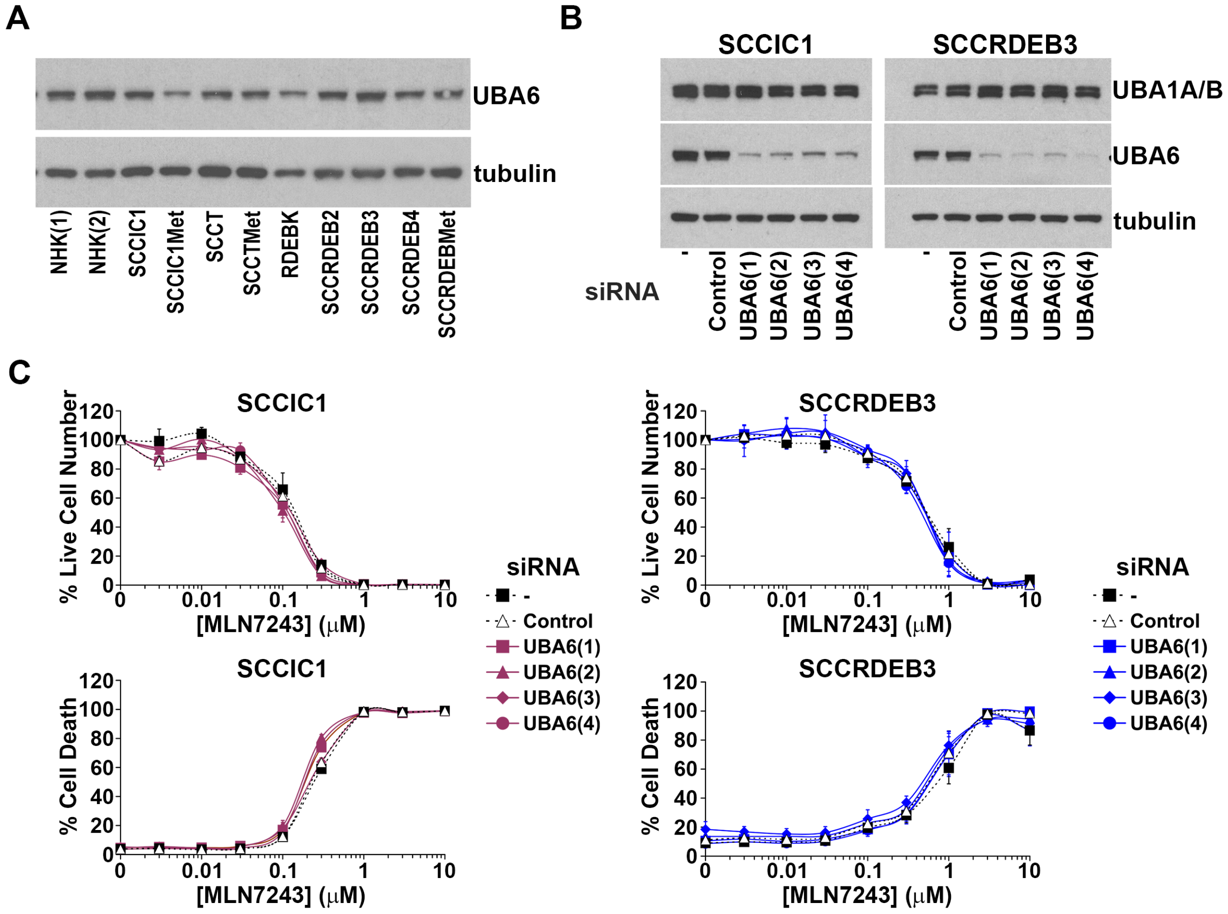

Supplementary Figure 8: Knockdown of the E1 UBA6 has no effect on the susceptibility of cSCC cells to MLN7243. **(A)** Protein expression of UBA6 in the panel of cell used in the study. Levels of UBA6 expression are relatively uniform. **(B)** MLN7243-resistant SCCIC1 and SCCRDEB3 cell lines were transfected with 4 individual siRNAs complementary to UBA6. Cells were harvested 72 hours after transfection. UBA6 protein expression was strongly reduced while levels of UBA1A and B were not changed. **(C)** SCCIC1 and SCCRDEB3 cells were transfected with UBA6 siRNAs and treated with MLN7243 for 72 hours. Cell viability (live cell number) expressed as a percentage of carrier alone for each siRNA and cell death were assessed by real-time imaging. Values are the mean -/+ SEM of 3 independent experiments. UBA6 depletion did not alter sensitivity to MLN7243.

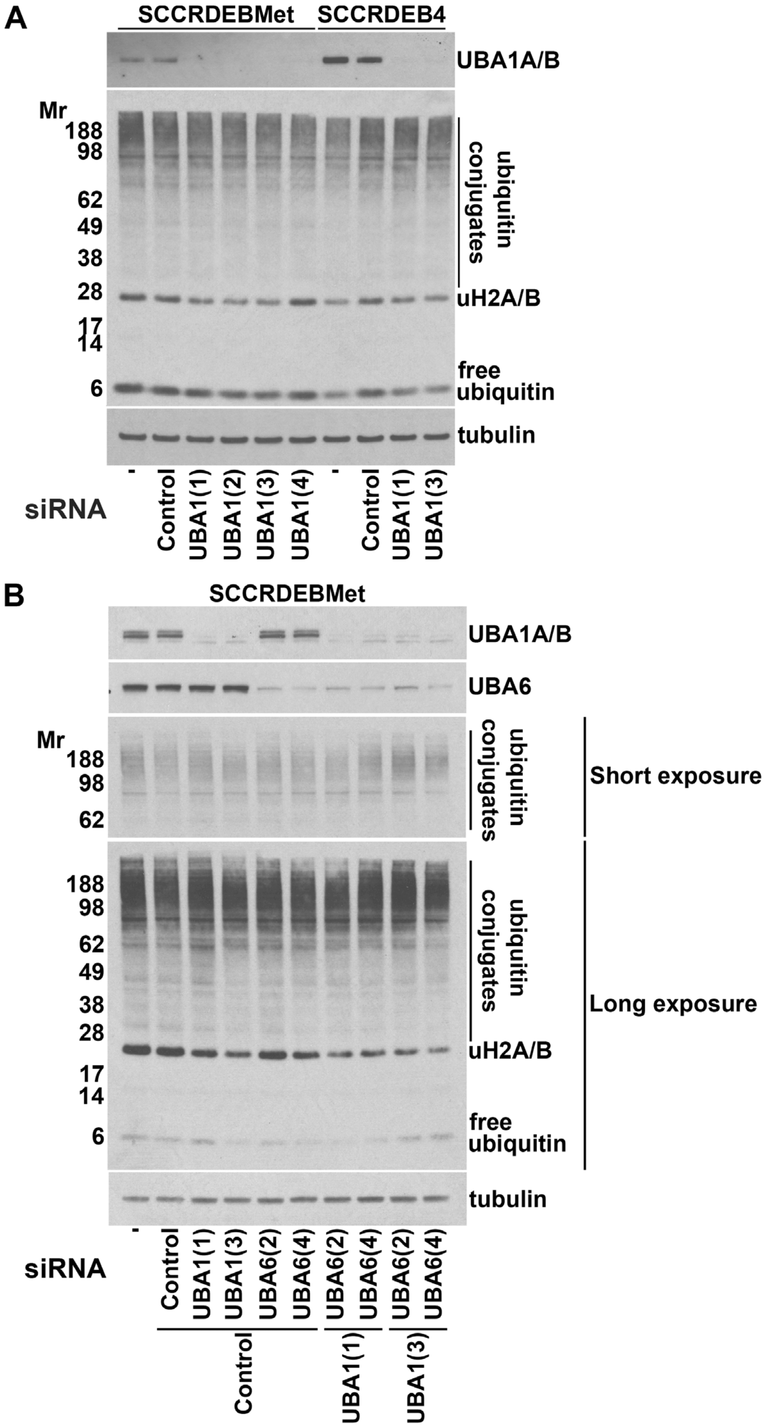

Supplementary Figure 9: Ubiquitin E1s are not rate-limiting for bulk ubiquitin conjugation in cSCC cells. **(A and B)** Cells were transfected with siRNAs targeting UBA1 and/or UBA6. The pattern of ubiquitination and ubiquitin E1 expression was analysed by western blotting 72 hours after transfection. (A) UBA1 knockdown had little effect on high molecular weight ubiquitin conjugates in the SCCRDEBMet and SCCRDEB4 cell lines. (B) Knockdown of UBA6 alone and in combination with UBA1 did not alter high molecular weight ubiquitin conjugates in SCCRDEBMet cells.

siRNAs (Dharmacon ON-TARGETplus)

BAK(1): J-003305-06, BAK(2): J-003305-07, BAK(3): J-003305-09
BAX(1): J-003308-11, BAX(2): J-003308-12, BAX(3): J-003308-13
BOK(1): J-004394-05, BOK(2): J-004394-06, BOK(3): J-004394-07
Control: D-001810-01
c-MYC(1): J-003282-25, c-MYC(2): J-003282-26
NOXA(1): J-005275-10, NOXA(2): J-005275-11, NOXA(3): J-005275-12
UBA1(1): J-004509-05, UBA1(2): J-004509-06, UBA1(3): J-004509-07, UBA1(4): J-004509-08
UBA6(1): J-006403-09, UBA6(2): J-006403-10, UBA6(3): J-006403-11, UBA6(4): J-006403-12

Antibodies

β-Actin (ab8226: Abcam, Cambridge, UK)
BAK D2D3 (6947P: New England Biolabs, Hitchin, UK)
BAX D2E11 (5023: New England Biolabs)
Cleaved Caspase-3 Asp175 5A1E (9664S: New England Biolabs)
eiF2α D7D3 (5324T: New England Biolabs)
P-eiF2α D9G8 (3398T: New England Biolabs)
c-MYC 9E10 (Prepared in house)
NOXA 114C307 (ab13654: Abcam)
PARP (9542S: New England Biolabs)
PSMB5 (PW8895: Enzo Life Sciences, Exeter, UK)
PSMC1 (11196-1-AP: Proteintech Group, Manchester, UK);
PSMD11 (14786-1- AP: Proteintech Group)
α-Tubulin DM1A (T9026: Sigma-Aldrich, Dorset, UK)
UBA1A (4890S: New England Biolabs)
UBA1A/B (4891S: New England Biolabs)
UBA6 (13386S: New England Biolabs)
UBE2Z (16928-1-AP: Proteintech Group)
Ubiquitin P4D1-A11 (05-944: Upstate, Temecula, CA, USA)

Supplementary Figure 10: siRNAs and primary antibodies used in this study.
